# Aging modulation of the immune system and immunotherapy efficacy in cancer

**DOI:** 10.3389/fimmu.2026.1781885

**Published:** 2026-03-11

**Authors:** Yurong Wang, Mengjie Liang, Yichen Mao, Wei Zhu, Xiaofei Shen, Wenxian Guan

**Affiliations:** 1 Department of General Surgery, Nanjing Drum Tower Hospital, Affiliated Hospital of Medical School, Nanjing University, Nanjing, China; 2 MOE Key Laboratory of Model Animal for Disease Study, Nanjing University Medical School, Nanjing, China; 3 Department of Anesthesiology, Jiangsu Cancer Hospital & Jiangsu Institute of Cancer Research & The Affiliated Cancer Hospital of Nanjing Medical University, Nanjing, China

**Keywords:** cancer immunotherapy, immunosenescence, older cancer patients, personalized medicine, tumor microenvironment

## Abstract

Immunosenescence is characterized by immune decline and chronic inflammation. With advancing age, the incidence of tumors increases significantly. Understanding how immunosenescence influences the initiation and progression of tumors, as well as its implications for tumor immunotherapy, has become a matter of urgent importance. This review begins with an analysis of the phenotypic changes and underlying mechanisms associated with immune system and immune cell aging, and further explores the interplay between immunosenescence and tumorigenesis. Evidence indicates that cytokines, cell interactions and other mediators serve as critical links connecting aging and cancer, exerting complex anti-tumor and pro-tumor effects. However, in the context of immunosenescence, these factors collectively contribute to the formation of an immunosuppressive tumor microenvironment (TME) that facilitates tumor immune evasion and proliferation. Clinical data reveal that immunotherapy in older adults is often challenged by variable treatment efficacy and reduced tolerance. This review systematically summarizes the data related to elderly patients in immunotherapy for different types of cancers, and discusses potential immunotherapy sensitization strategies tailored for elderly patients and the mechanisms and immunomodulatory effects of senescence-modulating drugs, with the aim of enhancing therapeutic response rates and improving safety profiles.

## 1 Introduction

The progression of aging induces not only normal physiological aging, but is also accompanied by a considerable array of pathological alterations following the decline of systemic functions. Factors such as the accumulation of intracellular DNA damage, diminished efficiency of cellular repair mechanisms, recurrent exposure to environmental triggers, metabolic syndrome, and a state of chronic inflammation, collectively contribute to an elevated risk of carcinogenesis (1). Among these alterations, immunosenescence stands out as a hallmark feature shaping the clinical trajectory of elderly cancer patients (2, 3). The majority of elderly patients are diagnosed at advanced stages of the disease (4), which results in poor clinical prognoses and low treatment tolerance (5). Concurrently, their distinct physiological characteristics, including multi-system functional decline and reduced drug metabolism, necessitate a multifaceted assessment prior to the selection of a clinical regimen, thereby increasing the therapeutic complexity (6, 7). In response to this phenomenon, this review explores the correlation between aging and tumors, revealing a bidirectional interaction between the two. In particular, it highlights how immunosenescence and chronic inflammation promote tumor initiation, progression, and metastasis in elderly patients, while also exerting a significant impact on their treatment outcomes and prognosis. These effects are mediated by multiple senescence-associated signaling pathways, involving factors such as senescent immune cells and dynamic cellular interactions within the aging tumor microenvironment (TME).

In recent years, the advent of cancer immunotherapy, exemplified by immune checkpoint inhibitors (ICIs) and chimeric antigen receptor T cell therapy (CAR-T), has revolutionized the clinical treatment paradigm. However, evidence indicates significant heterogeneity in the response rates of elderly patients to ICIs, with the underlying mechanisms remaining unelucidated. The underrepresentation of older patients in some clinical trials has resulted in divergent data regarding efficacy and safety in this cohort (7). Based on this, this review synthesizes aging-related immunotherapy data and reveals that the impact of age on immunotherapy efficacy varies across different cancer types. Additionally, it summarizes senescence-modulating drugs, evaluating their therapeutic benefits and potential limitations in the context of immunotherapy. Through this analysis, the review explores tailored immunotherapeutic strategies for the elderly population.

## 2 Aging and immunosenescence

With advancing organismal age, the functionality of the immune system declines—a process termed immunosenescence—which is characterized by an impaired capacity to clear deleterious substances and a concomitant increase in excessive inflammatory responses. This process can perpetuate a vicious cycle of aging and predispose individuals to autoinflammatory and autoimmune disorders (2, 3). Immunosenescence dysregulates both the innate and adaptive arms of the immune system. In the innate immunity system, the components exhibit functional deficits, including reduced formation, phagocytic activity, and chemotaxis of neutrophils, alongside their increased susceptibility to apoptosis (8). In the adaptive immune system, thymic involution leads to a reduction in the naive T-cell pool, decreased T-cell receptor (TCR) diversity, and an imbalance in memory T-cell populations, which collectively impair antigen recognition capabilities (9). B-cell antibody affinity and the durability of the adaptive immune response are also reduced (10). The expansion of memory cells results in immunological resource depletion, and different antigens cause the upregulation of pro-inflammatory molecules (11, 12). This culminates in a comprehensive decline in immune function, which not only weakens host defense against pathogens but also intensifies chronic inflammation, impairs immune surveillance, and results in an elevated risk of infection and malignancy (3, 13).

Immunosenescence leads to a dysregulation of inflammatory homeostasis within the immune system, preventing the effective clearance of senescent cells and pro-inflammatory factors. This culminates in a systemic, low-grade inflammatory state known as “inflammaging” (14). In the absence of overt infection, this condition is also termed “sterile inflammation”. It is distinct from acute inflammation and is characterized as a low-grade, systemic, and persistent inflammatory state associated with elevated levels of inflammatory biomarkers such as interleukin-6 (IL-6), interleukin-1 (IL-1), tumor necrosis factor-alpha (TNF-α), and C-reactive protein (CRP). These age-related immunological impairments and the resultant inflammatory state shift the body towards a catabolic metabolism and are correlated with a spectrum of diseases, frailty, and mortality (15, 16).

### 2.1 Immunosenescence: immune cell-specific features and functional impacts

**Figure 1:**
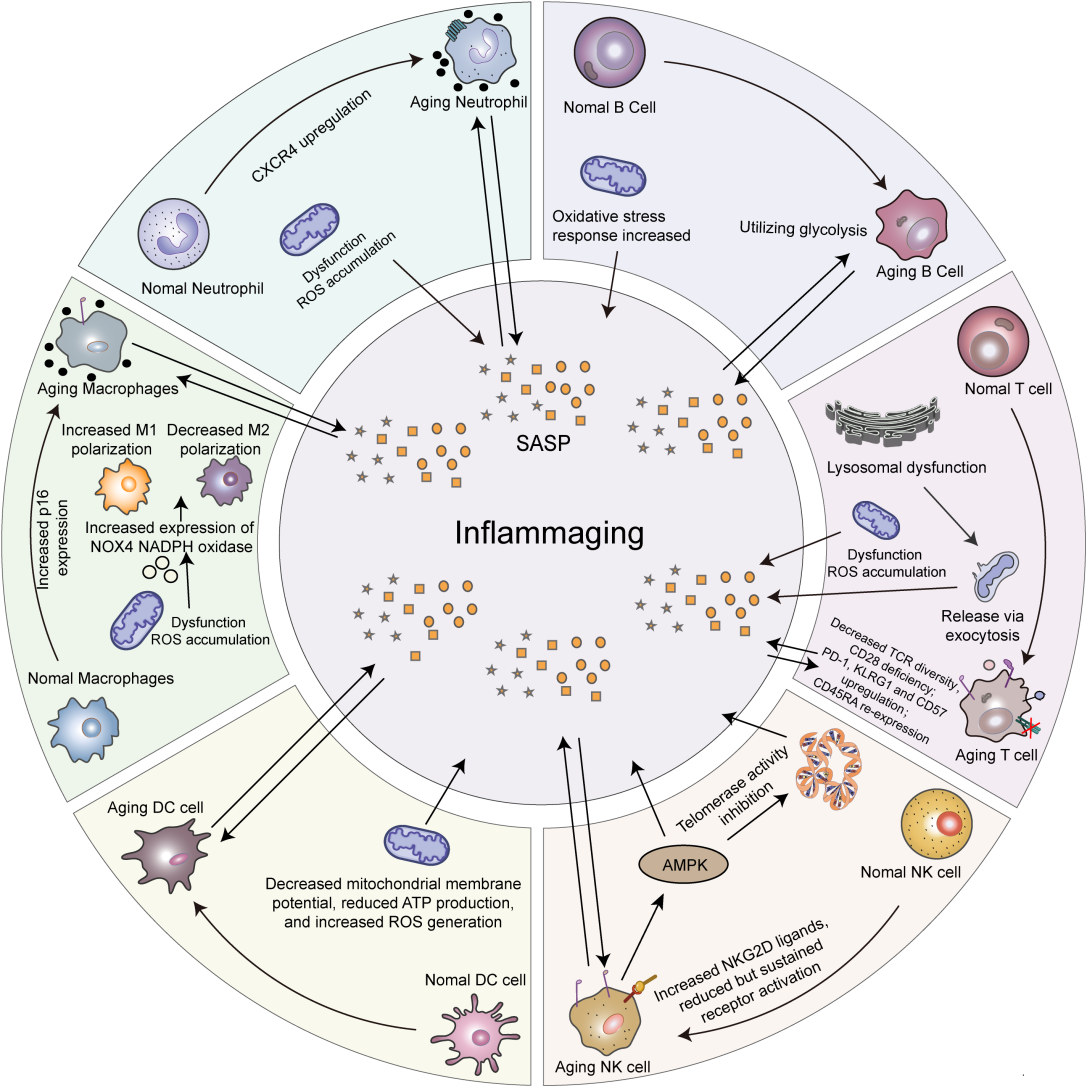
Changes related to immune cell senescence. Aging is associated with alterations in both innate and adaptive immune cells, resulting in increased levels of senescence-associated secretory phenotype (SASP). Neutrophil senescence is marked by elevated mitochondrial reactive oxygen species (ROS) and a significant upregulation of CXCR4 (C-X-C motif chemokine receptor 4), which directs bone marrow homing for clearance and serves as a key senescent marker. Macrophage senescence is defined by upregulated p16, mitochondrial dysfunction/ROS, and increased NADPH oxidase 4 (NOX4) expression, leading to M1-skewed polarization. This results in impaired phagocytosis, defective debris clearance, and chronic inflammation. Senescent DCs show reduced mitochondrial membrane potential, ATP, and increased ROS. NK cell senescence features a shifted receptor profile and activation of the killer cell lectin-like receptor subfamily G member 1 (KLRG1) -AMP-activated protein kinase (AMPK) pathway, driving functional decline. Senescent T cells are defined by the CD28^-^KLRG1^+^CD45RA^+^ phenotype (cluster of differentiation 28 negative, KLRG1 positive, cluster of differentiation 45 RA isoform positive); this is accompanied by mitochondrial dysfunction and consequent ROS accumulation. B cell aging is marked by a metabolic shift toward glycolysis and elevated mitochondrial oxidative stress. These immunological changes contribute to the development of chronic low-grade inflammation, often referred to as “inflammaging”, which is linked to multi-system dysfunction and the onset of age-related disease.

As shown in **Figure 1**, all immune cell lineages are impacted by aging to varying degrees, which compromises the capacity of the immune system to respond to novel antigens. Within the innate immune system, the phagocytic function of neutrophils and macrophages is attenuated, the antigen-presenting capacity of cells such as dendritic cells (DCs) is diminished, and the function of natural killer (NK) cells becomes dysregulated (8). Neutrophil Senescence is characterized by increased mitochondrial reactive oxygen species (ROS) and significantly elevated C-X-C motif chemokine ligand 4 (CXCR4) levels; the latter mediates neutrophil homing to the bone marrow for clearance and serves as a distinct marker of neutrophil senescence (17). Macrophages Senescence is characterized by elevated levels of p16 and SA-β-Gal, concurrent with mitochondrial dysfunction and increased ROS production; this subsequently leads to increased expression of NOX4 NADPH oxidase, resulting in polarization imbalance (18).Macrophages display attenuated phagocytic capacity, impaired debris clearance and polarize towards a CD80^+^ pro-inflammatory M1 phenotype, contributing to a chronic inflammatory state (19, 20). Senescent DCs exhibit reduced mitochondrial membrane potential, decreased ATP generation, and increased production of ROS. Mitochondrial dysfunction not only impairs phagocytic capacity, but the accumulation of ROS also further interferes with the process of antigen cross-presentation (18). Senescence in NK cells manifests as alterations in surface markers and functional impairment, characterized by the downregulated expression of activating receptors (NK group 2 member D (NKG2D), NKp30), the upregulated expression of inhibitory receptors (KIRs, NKG2A), and the activation of the killer cell lectin-like receptor subfamily G member 1 (KLRG1) -AMP-activated protein kinase (AMPK) signaling pathway (21). Furthermore, NK cells function is compromised, manifesting as impaired immune surveillance, a shift towards a more mature cytotoxic phenotype (CD56dim NK cells), and reduced cytokine secretion (CD56Bright NK cells). This collectively leads to attenuated immune surveillance and the occurrence of immune escape (the ability of tumor cells to avoid recognition and elimination by the immune system) (22).

In the adaptive immune system, B cells from elderly individuals demonstrate an enhanced ability to shift from oxidative phosphorylation to anaerobic glycolysis, accompanied by increased mitochondrial oxidative stress. This leads to a state of oxidative damage and cellular senescence, a metabolic reprogramming that parallels features observed in tumor cells (23). Romero et al. demonstrated that co-culturing B cells from elderly donors with normal CD4^+^ T cells induced the expression of inflammation-associated markers and increased lactate production in the T cells (23).

Senescent T cells are characterized by the loss of CD28, the upregulation of KLRG1, and the re-expression of CD45RA, accompanied by mitochondrial dysfunction that leads to the accumulation of ROS (24).T cells participate in inflammaging through functional dysregulation—including an expansion of pro-inflammatory subsets, a reduction in anti-inflammatory functions, and the secretion of pro-inflammatory cytokines (13, 25)—and further exacerbate the chronic inflammatory milieu through interactions with the Senescence-Associated Secretory Phenotype (SASP). SASP is composed of pro-inflammatory factors such as IL-6, IL-1, and TNF-α secreted by senescent cells, which can induce senescence in adjacent cells through paracrine signaling, thus establishing and intensifying a chronic inflammatory environment and accelerating systemic aging and age-related pathologies (26, 27). Research indicates that activated T cells in the elderly exhibit lysosomal dysfunction, which prevents the effective degradation of damaged mitochondria. Instead, these cells release necrotic mitochondria and their DNA via exocytosis, a process that contributes to inflammaging (28). Desdín-Micó et al. identified T cells deficient in mitochondrial transcription factor A as accelerators of immune system aging (29), while Ovadya et al. found that perforin gene knockout resulted in a more rapid accumulation of senescent cells across multiple murine organs, thereby accelerating aging (30). Elevated expression of the E3 ubiquitin ligase bifunctional apoptosis regulator (BFAR) in aged individuals inhibits the generation of tissue-resident memory T cells (TRM), thereby impairing the differentiation of CD8^+^ T cells into the TRM lineage (31). This impairs the capacity of T cells to recognize and eliminate novel tumor antigens.

## 3 Aging and cancer

In China, the age-specific incidence of all cancers is relatively low in the 0–34 age group, increases markedly from the 35–39 age group, and culminates in a peak in the 80–84 age group (32). Aging and cancer share numerous characteristics and exhibit a strong degree of equivalence (33). Concurrently, the state of chronic, low-grade inflammation inherent in elderly individuals exerts a pro-tumoral effect (34). Elevated levels of various inflammatory cytokines are known to have a detrimental impact on anti-tumor immunity (27).

### 3.1 Aging and the tumor microenvironment

With advancing age, the tissue microenvironment—comprising the extracellular matrix (ECM), immune cells, fibroblasts, and the vascular system—undergoes functional alterations.

ECM remodeling is a recognized hallmark of cancer, driven predominantly by the interplay between immune cells and cancer-associated fibroblasts (CAFs) (35). This process leads to the accumulation and increased cross-linking of non-cellular components such as collagen and elastin, which in turn alters tissue stiffness and mechanical stress (36). These changes promote tumorigenesis and establish a physical barrier that impedes drug delivery and immune cell infiltration, thereby fostering an immunosuppressive state (37). Tumor tissue often exhibits increased stiffness. Cells sense these physical properties of the ECM via integrin-conjugated focal adhesions, which influences cell migration, differentiation, and survival. Meanwhile, the remodeled ECM directly promotes tumor cell invasion and metastasis (38).

**Figure 2:**
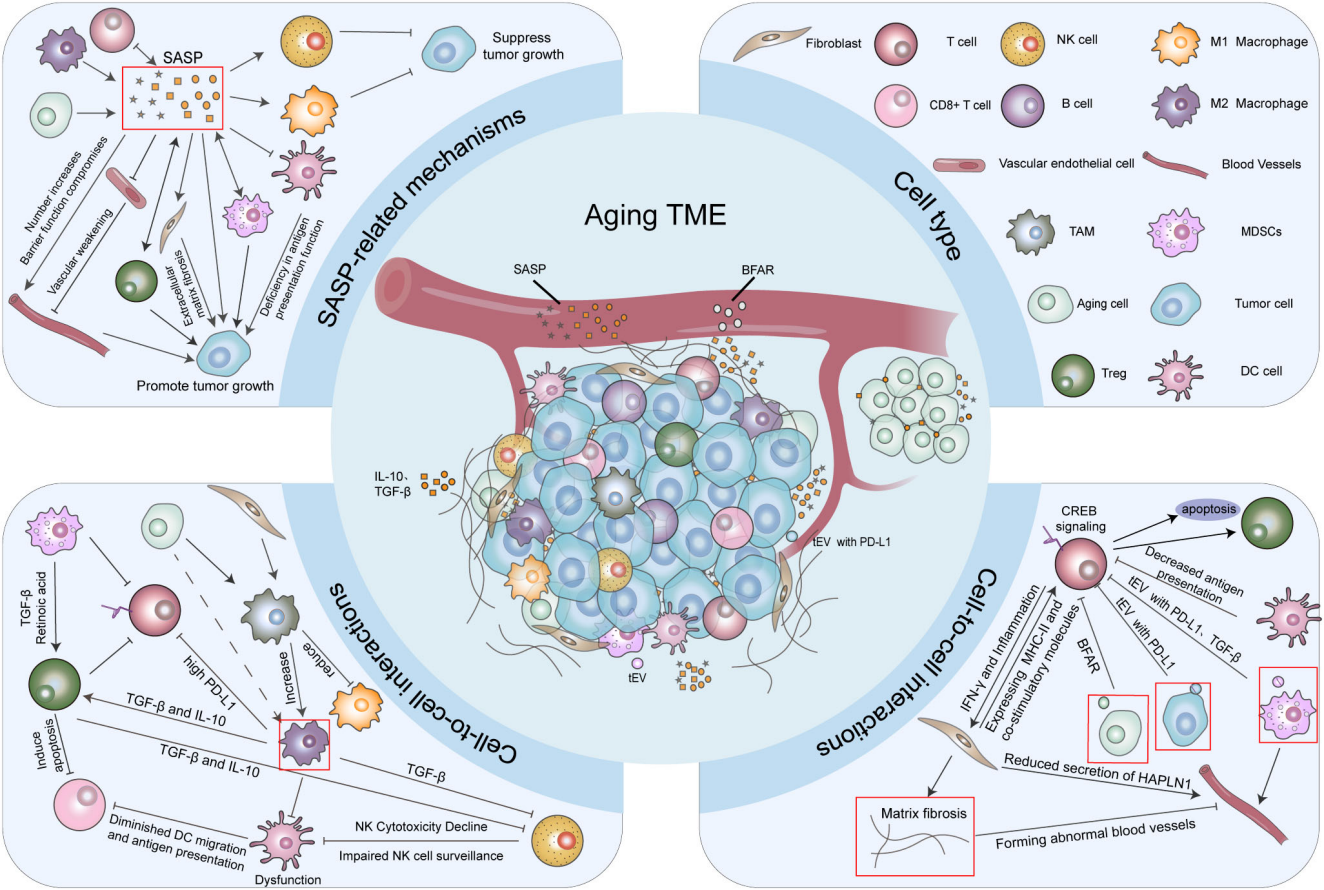
The changes in the tumor microenvironment caused by aging. In aging tumor microenvironment (TME), senescent cells, M2-type macrophages, T cells, myeloid-derived suppressor cells (MDSCs) and regulatory T cells (Tregs) mainly secrete the senescence-associated secretory phenotype (SASP), which activates fibroblasts and promotes the epithelial-mesenchymal transition of tumor cells. Simultaneously, SASP inhibits T cell function. Through interactions with SASP, T cells induce further cellular senescence and cytokine release, resulting in fibroblast dysfunction and pathological fibrosis of the matrix. SASP causes the weakening of vascular walls, which increasing tumor vessel density but compromises barrier function. Meanwhile, SASP recruits MDSCs and Tregs, suppresses dendritic cell (DC) function, and exerts immunosuppressive effects. However, SASP can also promote M1 macrophage differentiation and natural killer (NK) cell function, thereby exerting anti-tumor effects. Aging cells and fibroblasts influence tumor-associated macrophages (TAMs), promoting their differentiation into the M2 phenotype. M2-type macrophages, in turn, activate Tregs and secrete immunosuppressive cytokines such as interleukin-10 (IL-10) and transforming growth factor-beta (TGF-β), which impair DCs and NK cells function. M2-type macrophages also upregulate PD-L1, thereby suppressing T cells. NK cells and DCs exhibit functional abnormalities due to alterations in signal transduction pathways, impairing their ability to activate CD8^+^ T cells and leading to suppressed T cell function. Additionally, tumor cells and MDSCs release extracellular vesicles containing PD-L1. PD-1/PD-L1 signaling drives immune evasion by inducing T cell apoptosis and expanding immunosuppressive Tregs. Senescent cells also secrete Bifunctional Apoptosis Regulator (BFAR), which suppresses the activity of CD8+ T cells. Some key items are circled in red.

As shown in **Figure 2**, the TME in older individuals is characterized by immune cell dysregulation. Aging impairs the infiltration and function of CD8^+^ T cells within the tumor. Recent studies indicate that tumor-derived extracellular vesicles (tEVs) carrying Programmed death-ligand 1 (PD-L1) can induce T-cell senescence and immunosuppression via alterations in lipid metabolism and cAMP response element-binding protein (CREB) signaling (39). And the Programmed cell death protein 1 (PD-1)/PD-L1 interaction facilitates immune evasion by directly inducing T cell apoptosis and promoting the expansion of immunosuppressive regulatory T cells (Tregs) (40). The TME of older patients exhibits alterations in the NK cell-DC-CD8^+^ T cell signaling axis. These alterations encompass compromised NK cell immune surveillance, attenuated antigen presentation by DCs, and intrinsic changes in T cells themselves (8, 41, 42), which collectively diminish the priming of T cells by conventional type 1 DCs (cDC1s). This specifically impacts the differentiation of CD8^+^ T cells, driving them into a state of tumor-infiltrating age-associated dysfunction that is functionally, transcriptionally, and epigenetically distinct from canonical exhaustion and persists within the TME (42). The age-associated decline in the migratory capacity of DCs further exacerbates T-cell functional deficiencies, promoting tumor development, immune escape, and a suboptimal response to ICIs. Improving cDC1s function through bone marrow-targeted immunotherapy can enhance CD8^+^ T-cell immunity in aged mice (42). Similarly, inducing a hyperactivated state in DCs with agonists such as PGPC can rectify their age-related defects and enable Type 1 T helper cells (Th1) to acquire cytotoxic potential, thereby exerting anti-tumor activity (43).

Senescent fibroblasts can modulate tumor-associated macrophages (TAMs) to express pro-tumorigenic growth factors (M2-type), which in turn suppress the function of tumor-related DCs via the secretion of immunosuppressive factors such as interleukin-10 (IL-10) and transforming growth factor-beta (TGF-β), induce a Th2-polarized immune response, and promote the generation of Tregs, thereby attenuating anti-tumor immune response (27, 44). In the elderly, age-related macrophage dysfunction leads to a diminished capacity for clearing damaged ECM components as well as promoting tissue repair, and an upregulation of KEGG gene sets associated with fibrotic signaling (8, 45, 46). Furthermore, a decline in T-cell function results in aberrant, cytokine-mediated regulation of fibroblast activity, leading to the abnormal secretion of non-cellular matrix components (47).In aged individuals, persistent low-grade inflammation gives rise to a fibrotic SASP (fibrosis-related components of the SASP), activates fibroblasts, impairs tissue regeneration and repair, and facilitates tumor progression by modulating cancer cell stemness and the epithelial-mesenchymal transition (EMT) through various signaling pathways and inflammatory mediators such as TGF-β (48). Tumors harbor distinct populations of CAFs, which can be activated by fibrotic SASP derived from aged individuals. Stromal CAFs are pivotal in tumor angiogenesis, whereas inflammatory CAFs (iCAFs) are implicated in the formation of an immunosuppressive microenvironment (37). CAFs release higher levels of SDF-1, which recruits endothelial progenitor cells into carcinomas, enhancing angiogenesis and thus tumor growth (49). Stromal fibrosis is associated with diminished therapeutic efficacy: tumors characterized by a high degree of fibrosis, such as pancreatic, hepatocellular, and breast cancers, exhibit poor responses to chemotherapy and immunotherapy (50, 51). In breast cancer patients undergoing anti-PD-1 immunotherapy, iCAFs demonstrate an enhanced propensity to promote cancer cell proliferation, EMT, and the establishment of an immunosuppressive microenvironment. Conversely, in melanoma, patients with high iCAF scores exhibit significantly prolonged overall survival (OS) and progression-free survival (PFS), coupled with higher objective response rates to both anti-PD-1 and anti- Cytotoxic T-lymphocyte-associated protein 4 (CTLA4) therapies (52).

SASP facilitates angiogenesis and tumor invasion by secreting matrix metalloproteinases (MMPs) to degrade ECM and releasing growth factors such as vascular endothelial growth factor (VEGF) and fibroblast growth factor (FGF) (53). Specifically, MMPs degrade elastin, causing the weakening of vascular walls (54). Myeloid-derived suppressor cells (MDSCs) promote EMT and angiogenesis by secreting IL-6, TGF-β, and EGF, and by producing large amounts of MMP-9 (55). In melanoma, the secretion of the ECM protein hyaluronan and proteoglycan link protein 1 (HAPLN1) by fibroblasts decreases with age. This indirectly upregulates intercellular adhesion molecule 1 (ICAM1) expression in vascular endothelial cells, leading to the phosphorylation and internalization of VE-cadherin. This process increases tumor vessel density but compromises barrier function, creating conditions for tumor cell entry into the bloodstream and distant metastasis, thereby promoting tumor progression (56).

### 3.2 Regulation of the tumor microenvironment by the SASP

#### 3.2.1 SASP

SASP is a secretory phenotype unique to senescent cells, characterized by the secretion of various proinflammatory cytokines (interleukins), chemokines (C-X-C motif chemokine ligand 1 (CXCL1), C-C motif chemokine ligand 8 (CCL8)), growth factors, ECM remodeling factors (matrix metalloproteinases), and non-protein components such as ROS and nitric oxide (NO) into the surrounding microenvironment, despite cell cycle arrest (57). SASP plays a dual role in tumor progression. In the early stages, it enhances immunosurveillance and suppresses tumorigenesis by recruiting M1 macrophages, Th1, and NK cells (mediated by factors like IL-1α, IL-6, and interleukin-8 (IL-8)). Conversely, in advanced stages and under conditions of chronic inflammation, it drives the formation of an immunosuppressive TME and facilitates the recruitment of MDSCs and Tregs. By releasing immunosuppressive cytokines (some of which overlap with SASP factors, such as TGF-β and IL-10), it inhibits T cell and NK cell functions, ultimately enhancing tumor invasion, metastasis, and therapeutic resistance (53, 58, 59).

A state of tumor-promoting inflammation (TPI), a chronic inflammatory state that facilitates tumor progression, is a recognized hallmark of cancer (60). Meanwhile, the chronic inflammatory state of elderly can exacerbate TPI and facilitates tumor progression. Senescent cells secrete transcription factors and inflammatory cytokines that play a critical role in promoting tumor proliferation, angiogenesis, and invasion/metastasis. For instance, chronic inflammation can exert a pro-tumoral effect by inhibiting the clearance of senescent and neoplastic cells by myeloid cells (61–63). Cytokines play dual roles in cancer, with interferons like Interferon-gamma (IFN-γ) exerting core anti-tumor effects, while sustained signaling can induce resistance. TGF-β is stage-dependent, suppressing early tumors but promoting progression later. Pro-inflammatory cytokines (IL-1, IL-6) drive tumorigenesis, whereas immunosuppressive IL-10 dampens immunity. Even potentially therapeutic cytokines like interleukin-18 (IL-18) are neutralized by endogenous inhibitors in the TME (63, 64). This section focuses on several representative SASP factors for discussion.

#### 3.2.2 IL-6

Elevated levels of IL-6 are considered a manifestation of primary aging and possess dual (65), context-dependent anti-tumor and pro-tumor functions. IL-6 has been observed to manifest in inflammatory sites, the TME, and during the process of aging. It has been demonstrated that IL-6 typically exerts a pro-carcinogenic effect by fostering chronic inflammation and activating pro-tumoral signaling cascades (66). High serum concentrations of IL-6 are an adverse prognostic indicator for a multitude of cancers (67).In the context of chronic inflammation and cancer, the signaling pathways associated with IL-6 are often constitutively activated (68). With respect to its anti-tumor activities, IL-6 released from skeletal muscle during physical exercise enhances insulin sensitivity in glycogen-storing tissues, stimulates the circulation of anti-inflammatory cytokines, mobilizes cytotoxic immune cells, and reduces DNA damage in cancer cells (69).

Supraphysiological levels of IL-6 are linked to T-cell immune dysregulation. Excess IL-6 can inhibit the *de novo* generation of induced Tregs from naive T cells—without affecting the development or function of natural Tregs—thereby diminishing anti-inflammatory capacity and promoting a pro-/anti-inflammatory imbalance that perpetuates a chronic inflammatory state (70, 71). The IL-6- janus kinase (JAK)- signal transducer and activator of transcription (STAT) 3 signaling pathway can promote tumor cell survival by inducing the expression of anti-apoptotic proteins (including Bcl-2, Bcl-xL, and survivin). It also enhances the expression of MMP-2, VEGF), and basic FGF (bFGF), thereby promoting tumor angiogenesis and metastasis (34, 72, 73). Furthermore, IL-6-dependent STAT3 signaling can drive the methylation and subsequent inactivation of the key tumor suppressor gene p53, which allows cancer cells to bypass critical cell cycle checkpoints and evade apoptosis induced by DNA damage (74, 75).

#### 3.2.3 IL-1, IL-17, and IL-22

IL-1α and IL-1β, known as alarmins, activate and amplify local inflammation via the IL-1 receptor (IL-1R). In aged individuals, the expression of SASP-associated factors, including IL-6 and IL-1β, is upregulated in renal tissue, with the quantity of IL-1β^+^ macrophages being substantially higher than in younger cohorts (46). Furthermore, immunosenescence enhances the tumor-induced myelopoietic response, leading to the local accumulation of myeloid progenitor-like cells within the TME. These cells produce copious amounts of IL-1α, which promotes tumor development (62).

IL-1 has long been implicated in inflammation-induced carcinogenesis. In a chronic inflammatory state, IL-1α and IL-1β can directly promote the generation of carcinogenic mediators such as nitric oxide and ROS (76, 77). IL-1 also acts on epithelial cells via IL-1R to directly facilitate malignant transformation mediated by the nuclear accumulation of NF-κB (78). Studies have shown that IL-1α-expressing monocyte-derived macrophages correlate with poorer survival and higher recurrence rates; disruption of IL-1R1 signaling early in tumorigenesis can normalize myelopoiesis and decelerate the growth of lung, colon, and pancreatic tumors (62). In addition to the intrinsic pro-tumoral effects of IL-1, the activation of IL-1 signaling in T cells promotes the secretion of interleukin-17 (IL-17) and interleukin-22 (IL-22) (78). IL-17 is involved in the pathogenesis of numerous cancers, including breast, liver, lung, and colon cancer, through multiple pathways (79–84). And the IL-17/IL-17RA signaling axis is also implicated in pancreatic cancer progression (85–87). IL-22, via the phosphorylation of STAT3, provides proliferative and migratory signals to transformed malignant cells and/or cells harboring oncogenic mutations (88).

#### 3.2.4 IL-18

In the context of cancer, IL-18 exhibits dual anti-tumor and pro-tumor activities. Its anti-tumor functions include enhancing NK cell activity, promoting the production of anti-tumorigenic IFN-γ, and modulating immune responses (89). Furthermore, when IL-18 is cleaved by caspase-3 within cancer cells, a 15 kDa truncated form, termed short IL-18, is generated. It translocates to the nucleus, where it facilitates STAT1 phosphorylation at serine 727 via CDK8 and enhances the expression and secretion of ISG15, and thereby mediate the clearance of various syngeneic tumors and colitis-associated colorectal cancer in murine models (90). Conversely, IL-18 can exert pro-tumor effects. The decoy receptor IL-18BP (IL-18 binding protein), which is abundantly secreted by cancer cells, binds competitively to IL-18, thereby limiting its anti-tumor activity in mice (91). Additionally, IL-18 can induce the phosphorylation of long-chain acyl-CoA synthetase 6 (ACSL6) at serine 674 (ACSL6 pS674) and its translocation to the cell membrane. ACSL6 pS674 can promote the stable formation of the IL-18 receptor (IL18R1-IL18 receptor accessory protein (RAP)) heterodimer, activating the IL-18R1/NF-κB signaling pathway and leading to the upregulation of various pro-cancer genes, ultimately promoting liver cancer growth and metastasis. The combination of knocking down ACSL6 or ACSL6 S674A with anti-PD-1 therapy can inhibit the recruitment of TAMs and neutrophils, significantly promote the recruitment and activation of CD8^+^ T cells, and enhance immunotherapy efficacy (92).In liver cancer and hematogenous metastases to the liver and lungs, NK cells have been found to highly express IL-1R8, which functions as a negative regulator of the IL-18 signaling pathway. By inhibiting the interaction between IL-18Rα and downstream signaling molecules (such as MyD88 and IRAK4), it attenuates NK cell activation, thereby exerting a pro-tumor effect (93).

#### 3.2.5 TNF-α and IFN-γ

IFN-γ and TNF-α released by the senescent microenvironment activate immunosuppressive networks while simultaneously promoting tumor proliferation through chronic inflammation (94); conversely, TNF-α and IFN-γ signaling can induce tumor cell senescence, apoptosis, and ferroptosis, aiding in tumor growth control (95). TNF-α exhibits dual antitumor and protumor effects. High doses can induce tumor necrosis (96) and are often used in recombinant form combined with chemotherapy for isolated limb perfusion (97); however, long-term, low-dose TNF-α promotes tumor development by inducing DNA damage and angiogenesis (96). In the senescent microenvironment, TNF-α secretion by TAMs, combined with oxidative stress in the TME, leads to reduced nuclear translocation of PPARγ and increased TNF-α secretion, further driving tumor progression (98). TNF-α upregulates PD-L1 expression via the NF-κB pathway, promoting immune escape (99), and enhances the invasive and metastatic capabilities of multiple cancers, including melanoma (98). TNF-α also participates in EMT via NF-κB, inducing tumor cell dedifferentiation and promoting tumorigenesis (100). *In vivo* experiments demonstrate that blocking the TNF/TNFR1 signaling pathway with targeted antibodies increases the proportion of melanoma-specific CD8+ T cells in the microenvironment and delays tumor growth (101).

IFN-γ is generally not classified as a typical SASP factor. However, within the senescent TME, it acts as a critical immunomodulator, serving as both an amplifier of SASP effects and an effector molecule in the immune system’s response to senescent cells (53). As a pivotal component in CAR-T therapy, IFN-γ activates macrophages, promotes their polarization toward the M1 phenotype, enhances T cell cytotoxic function, and improves the organism’s immune response to tumors (102). Conversely, IFN-γ can upregulate PD-L1 via the STAT1/IRF-1 axis, promoting immune escape (53). Upon stimulation with IFN-γ, fibroblasts are induced to express high levels of major histocompatibility complex (MHC) class II and costimulatory molecules (e.g., CD40, CD80/86). This enables them to directly present antigens to T cells and trigger their reactivation, thereby establishing a positive feedback loop that amplifies and sustains inflammatory and fibrotic responses (103).

### 3.3 Aging and immune surveillance of tumors

In tumor immunology, the immune system performs immune surveillance to recognize and eliminate nascent transformed cells. The concept of tumor immune surveillance is based on three principles (1): cancer cells possess antigenicity (2); cancer cells can be destroyed by the host’s immune response (through mechanisms analogous to those in tissue or organ transplant rejection); and (3) immunosuppression is correlated with a higher incidence of tumors (104). The relevant antigens are typically tumor-associated antigens and neoantigens, which are presented to T cells via MHC/human leukocyte antigen (HLA) molecules (105). Immune surveillance imposes a selective pressure on tumors, driving continuous mutation and a process known as immunoediting. This is characterized by a dynamic equilibrium where tumor cells generate new mutations, selecting for those with lower immunogenicity, while a competent immune system continuously recognizes and eliminates these mutated cells. If mutated cells successfully evade immune-mediated clearance, immune escape occurs. This refers specifically to the acquisition by tumor cells, through genetic or epigenetic alterations, of insensitivity to immune recognition and/or elimination, allowing them to gain a clonal advantage and form a clinically detectable tumor (106). Immunosenescence and chronic inflammatory states are mutually influential with cancer. Key senescence-associated signaling pathways in tumors include the following: Constitutive activation of the NF-κB pathway drives the transcription of proinflammatory genes while inhibiting autophagy and apoptosis; this upregulation of anti-apoptotic proteins leads to the accumulation of senescent cells (65, 107, 108). Hyperactive nutrient sensing via the mTORC1 pathway increases ULK1 phosphorylation and blocks autophagy, resulting in anabolic imbalance and T cell exhaustion (109, 110). Chronic activation of the JAK-STAT pathway drives cytokine storms, causes hematopoietic stem cell (HSC) dysfunction, and leads to T cell reduction (111, 112). The cGAS-STING pathway, activated by damaged cytosolic DNA, induces NF-κB-mediated inflammation but exhibits impaired type I interferon (IFN-I) production, resulting in weakened antiviral responses (113–115). Blunted energy sensing in the AMPK pathway results in a failure of mTOR inhibition and obstructed autophagy, causing the uncontrolled expansion of MDSCs and impaired T cell memory formation (116–118). Decreased melatonin secretion diminishes its triple protective effects: inhibition of NF-κB, scavenging of ROS, and optimization of mitophagy (119, 120). Downregulation of Sirtuin family members (SIRT1/3/6) elevates mitochondrial oxidative stress and reduces histone deacetylation, resulting in epigenetic dysregulation (121–123). Concurrently, the synergistic interaction between the NF-κB and STAT pathways sustains a chronic inflammatory state within the TME, thereby enhancing tumor malignancy (124). For example, the chronic inflammation associated with inflammaging can promote cancer initiation and progression by activating STAT5-related pathways, which enhance cell proliferation, expand the cancer stem cell population, and confer resistance to chemotherapy and EMT (48, 125). TNF-α stimulation induces the expression of MMP-9, which, upon activation, acts on numerous inflammatory substrates (126), leading to ECM degradation and remodeling and promoting tumor metastasis (127). Concurrently, various oncogenes are involved in coordinating inflammatory transcriptional programs—including RAS, RAF, protein tyrosine kinases, tumor suppressor proteins, and transcription factors—and the inflammatory responses they mediate are linked to angiogenesis and the recruitment of myeloid-derived monocytic cells (63).

The senescence process partially overlaps with tumorigenesis, as immunosenescence involves the continuous deposition of inflammatory factors and senescent cells (128). Senescent cells activate chronic inflammation via SASP, creating an inflammatory microenvironment (27) that recruits Tregs and MDSCs. This further induces effector T cell senescence and suppresses CD8^+^ T cell function (129, 130). NKG2D ligands released by senescent cells inactivate NK cells, forming a malignant “immune clearance-escape” cycle (131). These processes ultimately lead to the accumulation of senescent cells within the TME, establishing an immunosuppressive microenvironment that accelerates cancer progression (132).

Immunosenescence leads to a dual escape from immunosurveillance: reduced CD8+ T cell cytotoxicity, failure in senescent cell clearance, decreased Tregs activity, and dysregulated immune tolerance (133–135). Increased release of soluble NKG2D ligands by senescent cells causes persistent receptor activation, ultimately leading to NK cell exhaustion and failure of immunosurveillance (131). Immunosenescence also drives resistance to immunotherapy; DCs senescence results in reduced IL-10: interleukin-15 (IL-15) and IFN-α levels, hindering NK cells activation (136). Elevated PD-1/T cell immunoglobulin and mucin domain-containing protein 3 (Tim-3) levels in senescent T cells attenuate immunotherapy responses. This ultimately leads to defective antigen presentation and a T cell exhaustion phenotype (137).

## 4 Aging and Immunotherapy

Immunotherapy has demonstrated substantial potential in clinical oncology research. However, its feasibility and efficacy in older patients remain critical clinical questions. The capacity of the older population to benefit from immunotherapy may be contingent upon factors such as tumor type.

As shown in **Table 1**, numerous studies have analyzed the efficacy of immunotherapy in the older population, but their conclusions are partially discrepant due to limitations such as sample or subgroup size, tumor heterogeneity, variable criteria for defining older cohorts, and differing observational endpoints. In 2019, a meta-analysis from the First Affiliated Hospital of China Medical University, encompassing 34 clinical trials on advanced cancer and over 20,000 elderly patients, evaluated data on ICI monotherapy and combination therapy. The findings indicated that while patients in the <65 years and ≥65 to <75 years age brackets derived an OS benefit compared to control groups, the OS benefit for patients ≥75 years was of a shorter duration than that of the control group. A PFS benefit was observed in the <65 and ≥65 age groups, but not in the <75 and ≥75 age groups (161). A meta-analysis by Kasherman et al., including 38 clinical trials on advanced cancer and 10,669 elderly patients over 65, concluded that following ICI treatment, both younger and older patients across different subgroups could achieve similar, age-independent OS benefits (162). A retrospective study by Corbaux et al. on cancer patients receiving ICI monotherapy reported no significant difference in OS or PFS between 150 patients ≥70 years and 185 patients <70 years across various metastatic tumor types (163).

**Table 1:** Data on various tumor immunotherapy studies related to age.

Tumor type	Year	Inclusion type	Population	Age	Medication	Conclusion	Ref
NSCLC*	2022	IIIB/C or IV	1208	<75 vs. ≥75	ICI monotherapy; vs. Platinum-based double-drug chemotherapy; vs. Non-platinum chemotherapy	ICI monotherapy improves survival, especially in younger patients.	(138)
	2018	/	245	<60 vs. 60–69 vs. 70–79 vs. ≥80	ICI monotherapy	similar OS and better PFS in age <80; shortest OS and PFS in age ≥80	(139)
	2022	IIIB/C or IV	53719	<55 vs. 55–64 vs. 65–75 vs. ≥75	ICI monotherapy vs. ICI combined with chemotherapy	older patients have lower OS and 2-year survival rate in both group.	(140)
	2016	PD-L1 positive (≥1%).	1034	Med^*^:63	Pembrolizumab (10 or 2 mg/kg) vs. Docetaxel	Pembrolizumab provided superior OS regardless of age and better tolerance in elderly patients	(141)
	2022	IIIB/C, IV or recurrence; PD-L1 TPS* ≥50%.	26	75-90(Med:78)	Pembrolizumab 200mg q3w.^*^	certain tolerability.	(142)
	2019	Locally advanced or metastatic;PD-L1 TPS* ≥1%.	1274	<65 vs.≥65	Pembrolizumab 200mg q3w. vs. Platinum-based chemotherapy	Better survival and lower incidence of adverse events in pembrolizumab group, regardless of age	(143)
	2022	Locally advanced or metastatic, III(unresectable)/IV NSCLC*	466	<65 vs. ≥65	Cemiplimab or Placebo with Chemotherapy	Better OS and PFS of all patients; good efficacy and tolerance in elderly patients.	(144)
	2017	Locally advanced or metastatic (stage IIIB or IV).	1225(ITT:850)	<65 vs. ≥65	Atezolizumab vs. Docetaxel	ICI has a better OS, especially in older patients (≥65)	(145)
	2021	Chemotherapy-naive metastatic (stage IV)	1202	<65 vs 65-74vs. 75-84vs. ≥85	ABCP vs. BCP and ACP vs. BCP	ABCP showed a better survival, particularly in patients under 65	(146)
	2019	/	2612	<75 vs. ≥75	Pembrolizumab vs. chemotherapy	Pembrolizumab has a better OS and safety in in elderly patients(≥75).	(147)
SCLC	2023	stage III/IV, without radical radiotherapy or postoperative recurrence	65	<70 vs. ≥70	Atezolizumab + carboplatin + etoposide	Excellent efficacy and acceptable toxicity in elderly patients	(148)
Upper gastrointestinal tract cancer	2022	HER2(-), unresectable advanced or recurrent GC or gastric-esophageal junction cancer	724	/	Nivolumab or Placebo + chemotherapy	Certain therapeutic advantages in patients of different ages.	(149)
	2021	untreated, inoperable, HER(-) GC, gastroesophageal junction cancer or esophageal adenocarcinoma	1581	<65 vs. ≥65	Nivolumab + Chemotherapy vs. Chemotherapy alone	Combination therapy had better survival and PFS, with acceptable safety, regardless of the age	(150)
	2017	Advanced or recurrent gastric adenocarcinoma or adenocarcinoma of the gastroesophageal junction	493	<65 vs. ≥65	Nivolumab vs Placebo	Nivolumab has shown survival benefits in patients of all age groups	(151)
Breast cancer	2020	untreated, non-metastatic TNBC	1174	<65 vs. ≥65	Pembrolizumab or Placebo + chemotherapy	Better pCR^*^, more significant in elder group.	(152)
Liver cancer	2022	Local advanced, metastatic or unresectable	501	<65 vs 65–74 vs ≥75	Atezolizumab +Bevacizumab vs. Sorafenib	Combination therapy has better efficacy and acceptable safety, even in elderly patients.	(153)
Kidney cancer	2019	Newly diagnosed or recurrent stage IV ccCRCC^*^	861	<65 vs. ≥65	Pembrolizumab +Axitinib vs Sunitinib	Lower risk of death and better PFS in combination therapy, regardless of age.	(154)
Melanoma	2018	advanced or metastatic	99	≥75	Pembrolizuma vs. Nivolumab vs. Ipilimumab	Efficacious and well-tolerated in elderly patients of ICIs, particularly PFS outcomes associated with pembrolizumab.	(155)
	2018	/	538	<62 vs. ≥62	Pembrolizumab;Some patients have previously received treatment with MAPKi	Better response to PD-1 inhibitors in age ≥62	(156)
	2016	metastatic	3	≥90	Ipilimumab vs. Nivolumab vs. Pembrolizumab	Older patients can tolerate and benefit from ICIs.	(157)
CRC	2022	MSI-H or dMMR in mCRC	307	Med:63	Pembrolizumab monotherapy vs. Chemotherapy	Better PFS, better objective response rate, and better safety than chemotherapy in patients with MSI-H/dMMR mCRC in all age.	(158)
	2023	dMMR- mCRC	41	<65 vs ≥65	Pembrolizumab monotherapy	Good efficacy and safety in older patients.	(159)
Prostate cancer	2025	mCRPC^*^ with disease progression after receiving second-generation ARPI treatment	1030	<65 vs ≥65	Pembrolizumab +Docetaxel vs. Placebo+ Docetaxel	No significantly better efficacy than placebo group. No difference among different age groups.	(160)

*PFS, Progression-Free Survival; OS, Overall survival; ICI, Immune Checkpoint Inhibitor; NSCLC, Non-Small Cell Lung Cancer; SCLS, small cell lung cancer; TPS, Tumor Proportion Score; ITT, Intention-to-treat population; MAPKi, MAPK inhibitors; MSI-H, Microsatellite instability high; dMMR, mismatch repair deficiency; CRC, Colorectal cancer; mCRC, metastatic colorectal cancer; CRPC, Castration-Resistant Prostate Cancer; ARPI, Androgen receptor pathway inhibitors; APC, Atezolizumab+Carboplatin+ Paclitaxel; ABCP, Atezolizumab+Bevacizumab+Carboplatin+ Paclitaxel; BCP, Bevacizumab+Carboplatin+ Paclitaxel; ccRCC, clear cell renal cell carcinoma; pCR, pathological complete response; q3w, every 3 weeks; q2w, every 2 weeks; Med, median; TNBC, triple-negative breast cancer; GC, gastric cancer.

### 4.1 Differential efficacy of immunotherapy in various cancers with aging

#### 4.1.1 Non-small cell lung cancer and small cell lung cancer

In non-small cell lung cancer (NSCLC), multiple clinical studies have demonstrated that, in both first-line and subsequent-line settings, ICI monotherapy improves PFS (138), objective response rate (ORR) (142), median OS (mOS) (142), and OS duration compared to standard paclitaxel-based or platinum-based chemotherapy (141, 143–146, 164, 165).

However, neither a study by Nokihara of 1,208 patients with locally advanced or metastatic NSCLC using a 75-year age cutoff (775 on standard chemotherapy, 463 on ICI monotherapy) nor other clinical studies using a 65-year cutoff found a significant difference in the efficacy of ICI monotherapy between elderly and non-elderly cohorts (138, 141, 143–145, 165). But a study by Lichtenstein et al. involving 245 NSCLC patients treated with PD-1 and PD-L1 inhibitors suggested a trend toward prolonged PFS with increasing patient age. The risk of disease progression or death was significantly lower for patients aged 70–79 compared to younger patients, but a decline in PFS was observed in patients over 80. Patients aged 60–69 exhibited the longest survival, followed by younger patients. The mortality risk for patients aged 80 or older was significantly higher than for their younger counterparts (139). Similarly, another study of 53,719 patients with advanced NSCLC revealed that despite similar rates of ICI utilization across age groups, survival benefits differed markedly: younger patients with advanced disease derived a clear benefit, whereas a significant benefit was not observed in patients aged 75 and older (140).

Notably, a meta-analysis of randomized controlled trials (RCTs) confirmed that the OS benefit from ICI monotherapy in elderly NSCLC patients (≥75 years) was comparable to that of the general population, and the degree of clinical benefit correlated with PD-L1 expression levels, with patients having a PD-L1 tumor proportion score ≥50% experiencing longer OS (147). However, the use of ICI combinations in NSCLC has yielded slightly different outcomes. One clinical trial indicated that although the proportion of middle-aged and elderly patients in the combination therapy arms was relatively small, the results did not suggest a diminished benefit from combination treatment (164). The IMpower150 study evaluated the efficacy of adding bevacizumab to an ICI-chemotherapy backbone in NSCLC. The results showed that, irrespective of PD-L1 expression, both the three-drug (atezolizumab, carboplatin, paclitaxel) and four-drug (plus bevacizumab) regimens conferred superior OS compared to the bevacizumab-carboplatin-paclitaxel regimen. However, patients ≥75 years did not derive a benefit from the four-drug regimen, a finding presumed to be related to poorer tolerability (146). For small cell lung cancer, Shiono et al. retrospectively analyzed the efficacy of atezolizumab combined with carboplatin and etoposide in 65 patients with extensive-stage disease. The cohort included 36 patients in the elderly group (70–89 years; median age: 74) and 29 in the non-elderly group (43–69 years; median age: 67). The results showed an ORR of 73.8%, with 80.5% in the elderly group, and 65.5% in the non-elderly group. No significant differences were observed in mPFS (5.5 vs. 4.9 months, p = 0.18) or mOS (15.4 vs. 15.9 months, p = 0.24) between the two groups (148).

#### 4.1.2 Digestive tract tumors

In colorectal cancer, older patients, particularly those over 75, exhibit a higher prevalence of tumors with a CpG island methylator phenotype (CIMP-high), microsatellite instability (MSI) or mismatch repair deficiency (dMMR), and v-Raf murine sarcoma viral oncogene homolog B (BRAF) mutations, which suggests a greater potential for benefit from immunotherapy (166). The KEYNOTE-177 study enrolled 307 patients with dMMR/MSI-H metastatic colorectal cancer (mCRC) for first-line therapy. After a median follow-up of 44.5 months, pembrolizumab demonstrated a significant improvement in ORR (43.8% vs. 33.1%) and PFS (16.5 vs. 8.2 months) compared to 5-FU-based chemotherapy. The median OS in the pembrolizumab arm exceeded 4 years, with a significantly reduced risk of death. A subgroup analysis stratified by age 70 (ratio of patients <70 to ≥70 was approximately 2.4:1) revealed no significant difference in clinical benefit between the two age groups (158, 167). Building on this, Saberzadeh-Ardestani et al. retrospectively analyzed the outcomes of ICI monotherapy in 41 similar patients aged >75: the ORR was 49%, disease control rate (DCR) 56%, complete response (CR) rate 32% (7 patients), median PFS 21 months, and OS 36 months, with the overall efficacy trend being consistent with KEYNOTE-177. However, subgroup analysis identified liver metastasis as a negative prognostic factor in this older cohort, with a median PFS of only 6 months (HR = 3.4); whether this phenomenon is also observed in non-older patients requires further investigation (159).

For gastric cancer, in the first-line treatment of human epidermal growth factor receptor 2 (HER2)-negative advanced disease, the ATTRACTION-4 study (N = 724 Asian patients) showed that nivolumab plus chemotherapy significantly reduced the risk of disease progression and prolonged PFS compared to placebo plus chemotherapy, although a statistically significant difference in OS was not achieved (149). The CheckMate 649 study (a global, multicenter trial with >1500 patients with HER2-negative gastric cancer, gastroesophageal junction cancer, or esophageal adenocarcinoma) confirmed that a nivolumab plus chemotherapy regimen conferred benefits in both PFS and OS (150). The discordance in OS results between these two RCTs remains incompletely understood but may be attributable to factors such as population heterogeneity and sample size. Subgroup analyses from both studies suggested that the clinical efficacy in the ≥65 years elderly subgroup was consistent with that of non-elderly patients. However, the subgroup with concomitant peritoneal metastases exhibited a poorer response to ICI-chemotherapy combinations (149). In the realm of HER2-positive advanced gastric cancer, the addition of pembrolizumab to the standard regimen of trastuzumab plus chemotherapy can further and significantly enhance the ORR (168). In the later-line setting for advanced gastric cancer, nivolumab significantly prolonged OS compared to placebo, irrespective of PD-L1 expression status, with no statistically significant difference in clinical benefit observed between elderly and non-elderly patients (151). A *post-hoc* analysis of the ATTRACTION-2 study data revealed that patients <60 years of age with peritoneal metastases or hyponatremia had a suboptimal clinical response to nivolumab. In contrast, patients ≥60 years of age without peritoneal metastases and with normal serum sodium levels still derived a high degree of clinical benefit (169). These findings, combined with the subgroup analysis from ATTRACTION-4, suggest that peritoneal metastasis in gastric cancer may be an independent negative prognostic factor for immunotherapy, while patient age itself is not the primary determinant of treatment efficacy.

#### 4.1.3 Other malignancies

For head and neck squamous cell carcinoma, older patients, particularly those with recurrent or metastatic disease, can derive benefit from immunotherapy. The Check-Mate 141 trial demonstrated that nivolumab significantly prolonged OS compared to conventional chemotherapy; however, immunosenescence may diminish ICI efficacy, and the survival benefit from PD-1 inhibitors may be attenuated in patients over 75 years of age (170).

In breast cancer, the KEYNOTE-522 study, which included an 11.8% cohort of elderly patients (≥65 years) with triple-negative breast cancer (TNBC), showed that the pathological complete response (pCR) rate was superior with neoadjuvant pembrolizumab plus chemotherapy compared to chemotherapy alone, but the study did not include a dedicated analysis for the older subgroup (152). Preclinical evidence suggests that high levels of tumor-infiltrating lymphocytes (TILs) may correlate with better outcomes from neoadjuvant immunotherapy in TNBC (171). However, the percentage of TILs is known to decrease with age in breast cancer patients, particularly those with TNBC (166). This may be a contributing factor to the diminished immunotherapy efficacy observed in some elderly patients, and targeting this mechanism could potentially improve therapeutic outcomes (172).

For tumors with limited responsiveness to chemotherapy, such as advanced hepatocellular carcinoma and metastatic clear cell renal carcinoma, ICI monotherapy or ICIs in combination with anti-angiogenic agents targeting VEGF/VEGFR has been established as a first-line recommendation (Level I) in national and international guidelines. Age-stratified data from pivotal studies indicate that the treatment response in elderly patients (≥75 years) is comparable to that of the overall population, with a favorable safety profile (153, 154).

In the context of metastatic melanoma, clinical observations suggest that older patients (>60 years) exhibit a more favorable response to PD-1 inhibitors than their younger counterparts, and even nonagenarians can achieve a clinical response to ICIs (155–157). The underlying mechanism may be attributed to the presence of an aging-enriched enterotype (E/AE) in older individuals, which is associated with improved ICIs outcomes. Microbiota derived from the E/AE enterotype have been shown to enhance sensitivity to anti-PD-1 therapy in mice and remodel the TME (173).

Prostate cancer, typically managed surgically, is characterized by a low tumor mutational burden and an immunosuppressive TME, which constrains the efficacy of ICIs and necessitates the development of novel therapeutic strategies to augment the immune response (174). For instance, in metastatic castration-resistant prostate cancer, docetaxel remains the standard of care, and studies have shown that the addition of pembrolizumab did not increase efficacy in patients who had previously received docetaxel (160).

### 4.2 Strategies to sensitize older individuals to immunotherapy

A significant body of current research is focused on addressing immunosenescence in the context of cancer.

The formation of tertiary lymphoid structures (TLS) is an antigen-dependent process driven by chronic inflammatory signals within non-lymphoid organs. Single-cell analyses have revealed significant heterogeneity in their cellular composition and phenotypic states, which underlies their seemingly contradictory roles in disease. While the presence of TLS is generally associated with favorable clinical outcomes in cancer and infections, it conversely constitutes a hallmark of more severe disease in autoimmune and chronic age-related inflammatory disorders, such as chronic kidney disease (175). Based on this, a key therapeutic strategy for elderly cancer patients is to harness TLS that form within the context of immune aging. This approach aims to counteract the adverse effects of SASP on immune cells, thereby enhancing TLS function and ultimately unleashing their anti-tumor potential. Pharmacologically blocking PD-L1 while simultaneously downregulating the expression of inflammatory cytokines (including TNF-α, IL-1β, and IL-6) can disrupt the immunosuppressive network within the TPI, enhance cytotoxic T-lymphocyte infiltration, and thereby amplify therapeutic efficacy (176). As summarized in **Table 2**, various pharmacological strategies targeting the senescence-associated microenvironment and tumor-related inflammation have been identified. These agents exert antitumor effects primarily through two core mechanisms. The first involves acting as senolytics, such as ABT-263/Navitoclax (211, 212). These agents eliminate senescent cells within the TME, thereby reversing immunosuppressive traits and restoring the effector function of CD8^+^ T cells (211) to overcome resistance to immunotherapy (211, 213). Second, these agents function as SASP modulators, such as metformin (177), rapamycin (178–180), and biologics targeting critical inflammatory cytokines, particularly IL-6/IL-1β, exemplified by tocilizumab (190–193) and IL-1R antagonists (194).By intervening in signaling pathways including AMPK/mTOR (177), p38 MAPK (182, 183), and NF-κB (184), they inhibit the secretion of protumorigenic inflammatory factors such as IL-6 (177–180, 182, 210) and TNF-α (178–181, 183) by senescent cells, effectively remodeling the immune microenvironment. Furthermore, inhibitors targeting inflammation-associated pathways, including the NLRP3 inflammasome (206–209), COX-2 (245–251), and JAK/STAT (219–223), as well as certain clinically established drugs such as aspirin (229–238), have demonstrated significant potential in modulating both innate and adaptive immunity and enhancing the efficacy of ICIs (200, 211, 212, 214, 260). Currently, while the vast majority of these studies remain in the preclinical phase, select agents such as Navitoclax (211, 212)and tocilizumab (190–193) have already exhibited promising clinical utility in clinical trial.

**Table 2:** Mechanisms of senescence-regulating drugs and their impact on anti-tumor and/or immunotherapy.

Drug/Agent	Tumor Type/Indication	Mechanism of Action & Target	Impact on Immune Cells	Impact on ICIs* & Clinical Stage	Ref
Regulators targeting the modulation of SASP					
Metformin	HNSCC*	Inhibits secretion of SASP factors (IL-6, IL-8, MCP-1, GRO) via the AMPK/mTOR pathway; delays tumor progression.	Enhances antitumor efficacy of CDK4/6 inhibitors; modulates the TME immune landscape.	Preclinical studies.	(177)
Rapamycin	Wilms’ Tumor, Melanoma	Inhibits mTOR pathway, reducing IL-6, IL-8, TNF-α, and IL-1α secretion; autophagy activator; combined with TLR4/9 agonists.	Restores hematopoietic stem cell regenerative capacity; enhances influenza vaccine efficacy; reverses immune senescence; improves PD-1+ T cell function; activates autophagy to potentiate antitumor effects via immune modulation.	Enhances antitumor effects when combined with TLR4/9 agonists. Preclinical studies.	(178–180)
Resveratrol	Colorectal Cancer, Lung Cancer	Inhibits MAPK pathway; reduces IL-6, IL-1β, and TNF-α secretion.	Inhibits SASP-associated protumorigenic effects in MRC5 fibroblasts.	Preclinical studies.	(181)
Echinacea Extract	NSCLC*	Inhibits MAPK pathway; reduces IL-6, IL-8, and IL-1α secretion.	Inhibits formation of the senescence-associated secretory phenotype (SASP).	Preclinical studies.	(182)
SB203580	NSCLC*	Inhibits p38 MAPK; reduces secretion of IL-6, IL-8, IL-10, TGF-β, and TNF-α.	Inhibits SASP formation; improves the immune microenvironment.	Preclinical studies.	(183)
BIRB796	Glioblastoma	Inhibits p38 MAPK pathway; reduces IL-6 secretion.	Inhibits SASP formation.	Preclinical studies.	(183)
SYQ-PA	Breast Cancer	Modulates NF-κB pathway via PPARγ inhibition; promotes macrophage polarization from M2 to M1 phenotype.	Enhances antitumor immunity; inhibits TNF-α secretion.	Preclinical studies.	(184)
Biological agents targeting key inflammatory factors and receptors					
Siltuximab (CNTO328)	CRPC*, Multiple Myeloma, Solid Tumors, Prostate Cancer, Metastatic RCC*	Monoclonal antibody targeting IL-6.	Reduces CRP* levels; inhibits IL-6 signaling pathway; stabilizes disease in some patients.	Clinical Phase II (NCT00433446, NCT00911859).	(185–189)
Tocilizumab	Lung Cancer, TNBC*, Recurrent Epithelial Ovarian Cancer	Monoclonal antibody targeting IL-6 receptor; blocks the IL-6/STAT3/C/EBP-IL-6 feed-forward loop.	Reduces STAT3 activation and IL-6 production in TAMs. Safe and effective when combined with interferon-α2b chemotherapy.	Clinical application (Cachexia treatment); Preclinical studies (TNBC, Lung adenocarcinoma); Clinical Phase I (Ovarian cancer).	(190–193)
IL-1R Antagonist	Pancreatic Cancer	Antagonizes IL-1 receptor; enhances chemotherapeutic efficacy.	Primarily acts by blocking IL-1 signaling (specific immune subsets not specified).	Preclinical studies.	(194)
Infliximab	Colon Cancer	Monoclonal antibody targeting TNF-α; blocks TNF-α/TNFR2 signaling. Enhances chemotherapy efficacy.	Reduces CCR8^+^ regulatory T cells, decreasing TME immunosuppression.	Clinical studies; Clinical Phase I/II (well-tolerated); Preclinical studies show enhanced anti-PD-1 efficacy.	(195–197)
Carlumab (CNTO 888)	Metastatic CRPC*	Monoclonal antibody targeting CCL2.	Transiently inhibits CCL2.	Clinical Phase I/II trials (did not show significant single-agent anticancer activity).	(198)
Pattern recognition receptors and agonists/inhibitors of inflammasomes					
TLR3 Agonist (ARNAX)		TLR3 agonist; promotes interferon transcriptional induction; enhances immunotherapy efficacy.	Promotes dendritic cell maturation; enhances T cell activation and proliferation.	Promotes immunotherapy efficacy; Clinical studies.	(199)
TLR7/8 Agonist Nanoparticles		Activates TLR7/8; promotes polarization of tumor-associated macrophages (TAMs) toward an antitumor phenotype.	Promotes TAM polarization to M1 (antitumor) phenotype.	Enhances cancer immunotherapy efficacy; Preclinical studies.	(200)
STING Agonists (DMXAA, cGAMP, ADU-S100, etc.)	Pancreatic, Breast, Melanoma, and other cancers	Activates cGAS-STING pathway; induces Type I interferon production; promotes antitumor immunity.	Promotes dendritic cell maturation; enhances activation and infiltration of T cells and NK cells; reprograms macrophages.	Enhances antitumor effects when combined with radiotherapy, chemotherapy, CAR-T*, or CTLA-4/PD-1 antibodies. Preclinical and early clinical studies.	(201–205)
Nigericin	Breast Cancer, Neuroblastoma	Activates NLRP3 inflammasome; controls tumor growth.	Regulates immune response via NLRP3 activation, likely influencing IL-1β and IL-18 secretion.		(206)
OLT1177	Melanoma	Inhibits NLRP3 inflammasome; inhibits tumor growth.	Limits expansion of myeloid-derived suppressor cells, likely by affecting IL-1β.	Potential synergy with ICIs*. Preclinical studies.	(207–209)
Aging Clearance Agents (Senolytics) and Targeted Delivery Systems					
Quercetin	Osteosarcoma	Inhibits SASP secretion (IL-6, IL-8, MMPs, IL-1α, CXCL12, VEGF). Induces senolysis in doxorubicin-induced senescent fibroblasts.	Attenuates protumorigenic effects of senescent fibroblasts.	Preclinical studies.	(210)
ABT-263 (Navitoclax)	Lung Cancer, Breast Cancer, Murine Colon Cancer (MC38), Lymphoma (EL4)	Inhibits anti-apoptotic proteins (Bcl-2, Bcl-xL, Bcl-w) to clear senescent cells. Specifically eliminates p16^+^/p21^+^ senescent cells; reverses senescence-induced immunosuppressive myeloid phenotypes; reduces p16/p21 expression in spleen and TME.	Restores CD8^+^ T cell proportion, accumulation, and activation (IFNγ^+^) within tumors; increases CD8^+^ T cell/CD11b^+^ myeloid cell ratio; abrogates myeloid cell-mediated suppression of CD8^+^ T cell proliferation.	Reverses immunotherapy resistance: Restores anti-PD-L1 efficacy in senescent mice; restores abscopal effects of “radiotherapy + anti-CTLA-4” therapy; significantly prolongs survival when combined with ICIs*. Clinical Stage: Preclinical studies (validated senolytic; entered human clinical trials).	(211, 212)
ABT-737/Navitoclax (Bcl-2 inhibitor)	NSCLC* (Kras ^G12V^ model)	Eliminates senescent cells; reduces SA-β-gal activity; decreases proliferation markers (Ki67, pRb); increases tumor cell apoptosis.	Immune cell changes not detailed.	Combined with cisplatin: significantly reduces tumor burden; decreases senescence and proliferation markers; improves therapeutic response.	(213)
Ra/Ba SAzyme (²²³Ra/Ba SAzyme)	Lung Cancer	Induces senescence and ROS generation, followed by senescent cell clearance via anti-PD-L1.	Induces senescence to enhance antitumor immunity; effectively clears senescent cells upon combination with anti-PD-L1, reducing recurrence risk.	Preclinical studies.	(214)
Targeting chemokine receptors and downstream signaling pathways					
PF-04136309	PDAC*	Small molecule CCR2 antagonist.	Reduces CCR2^+^ monocytes and TAMs; alleviates immunosuppression; stimulates T cell infiltration into the TME.	Clinical Phase Ib trial (combined with chemotherapy; well-tolerated; showed early antitumor activity).	(215, 216)
BMS-813,160	Pancreatic, colorectal cancer(combination with chemotherapy or nivolumab)	CCR2 inhibitor.	Disrupts prosurvival microenvironment driven by CCL2-mediated monocyte and macrophage migration.	Preclinical studies	(217, 218)
Galunisertib (TGFBR1 Inhibitor)	NSCLC*, HGSOC*	Inhibits TGFβ/TGFBR1 axis; blocks AKT/mTOR pathway activation; reduces p-AKT/p-p70S6K levels; attenuates tumor cell proliferation, migration, and sphere-forming capacity.	No significant changes in immune cell proportions (except normalization of alveolar macrophages to pre-cisplatin levels); exhibits no significant immunosuppressive activity.	Combined with cisplatin: significantly reduces tumor burden; prolongs survival (median survival increased by 33–42.7%); improves weight maintenance and tolerability.	(213)
JAK-STAT signaling pathway inhibitor					
Bazedoxifene	Pancreatic Cancer, Breast Cancer	SERM*; effectively blocks IL-6/GP130-mediated STAT3 activation.		Preclinical studies (demonstrates repurposing potential in malignancies driven by aberrant IL-6 signaling).	(219)
Stattic	Prostate Cancer	STAT3 inhibitor.		Preclinical studies (*in vitro*, combined with Tocilizumab).	(220)
Ruxolitinib	aCML*, CNL*, Inflammation-related tumors	JAK1/JAK2 inhibitor; blocks JAK/STAT3 signaling; inhibits CRP* expression.	Significantly reduces inflammation-associated symptoms (e.g., fever).	Clinical studies (effective in patients with CSF3R mutations); Preclinical studies (demonstrates anti-inflammatory effects).	(221, 222)
AZD1480	HPV-related HNSCC*	JAK1/JAK2 inhibitor; targets JAK/STAT signaling to overcome cisplatin resistance.	Reduces protumor inflammation and immune evasion.	Preclinical studies (animal models).	(223)
Momelotinib	NSCLC*	JAK1/JAK2 inhibitor; combined with EGFR inhibitor Erlotinib.	Targets JAK/STAT-mediated inflammation and resistance pathways to delay or overcome EGFR inhibitor resistance.	Clinical Phase Ib study (safe and tolerable).	(224)
Tofacitinib	Inflammatory diseases (in context of cancer risk)	JAK1/JAK3 inhibitor; inhibits proinflammatory cytokines (IL-2, IL-6, IL-12, IL-23).	Inhibits immune system activation; may hinder immune surveillance of nascent tumor cells.	Systematic review and Meta-analysis (associated with slightly increased overall cancer risk).	(225)
Jaktinib	Myelofibrosis	Novel JAK inhibitor.		Clinical Phase II study (shows promise in patients relapsed or refractory to Ruxolitinib).	(226)
Pacritinib	Metastatic Colorectal Cancer, AML*	JAK2/FLT3 inhibitor.	Inhibits STAT3 signaling and proinflammatory cytokine levels.	Clinical studies (limited clinical activity in heavily pretreated patients); Preclinical studies (synergistic with HDAC inhibitors).	(227, 228)
COX inhibitors and non-steroidal anti-inflammatory drugs					
Aspirin	Colorectal, Breast, Pancreatic, Liver, and other cancers	Irreversibly acetylates and inactivates COX; inhibits multiple pathways (PIK3CA/AKT/PTEN, Wnt-β catenin, NF-κB); suppresses TGF-β, IL-6, MCP-1; regulates angiogenic/inflammatory cytokines.	Modulates T cell and macrophage responses; activates resolution pathways; promotes M1 macrophage polarization and inhibits M2 polarization, thereby limiting tumor progression.	Efficacy combined with ICIs* (e.g., pembrolizumab/ipilimumab) is comparable to ICIs* monotherapy. Epidemiological studies, clinical prevention (USPSTF recommended), and Clinical Phase II trials.	(229–238)
Sulindac	Colon, Laryngeal Cancer, etc.	Downregulates Sp proteins; induces apoptosis; inhibits Wnt/β-catenin pathway.	Reduces Fas ligand expression; may affect lymphocyte apoptosis.		(239–244)
Celecoxib	Colorectal, Breast, Pancreatic Cancer, etc.	Selective COX-2 inhibitor; inhibits Wnt/β-catenin signaling; induces apoptosis; inhibits EMT and metastasis. Suppresses tumor-derived PGE2; enhances chemotherapy (paclitaxel)-induced immunogenic cell death.	Inhibits IFN-γ-induced surface PD-L1 expression.	Enhances antitumor effects when combined with p65miRNA, statins, or TRAIL. Preclinical and clinical studies.	(245–251)
Ibuprofen	Colorectal, NSCLC*, Prostate, Liver Cancer, etc.	Inhibits COX-2; reduces expression of HDACs and histone demethylases; regulates β-catenin signaling.	May regulate the inflammatory microenvironment by inhibiting COX-2-mediated PGE2 production.	Preclinical studies.	(252–257)
Other drugs with anti-inflammatory or immune-regulating effects					
Silybum marianum (Silymarin) Flower Extract	Skin Cells	Inhibits IL-6 and MMP-1 expression.	Demonstrates anti-aging activity in human skin cells.	Preclinical studies.	(258)
CD38 Blockers		Blocks CD38; reduces intratumoral adenosine accumulation; restores CD8^+^ T cell activity.	Relieves CD38-mediated suppression of CD8^+^ T cells.	Sensitizes tumors to anti-PD-L1 therapy. Preclinical studies.	(259)
A2A Adenosine Receptor Inhibitors	Breast Cancer, Colon Cancer	Inhibits A2A adenosine receptor.	Promotes T cell proliferation; generates T cell-dependent antitumor activity.	Enhances anti-PD-1 efficacy. Preclinical studies.	(260)
Small Molecule OmoMYC	AML*	MYC-inhibiting peptide/miniprotein.	Increases immune cell infiltration into the tumor.	Sensitizes tumors to anti-PD-1 immunotherapy; currently in clinical development. Preclinical/Clinical trial development.	(261, 262)
Dexamethasone	Lymphoma, Breast, Pancreatic Cancer	Glucocorticoid (adjuvant for lymphoma); may promote progression in pancreatic/breast cancer. Combined with Tamoxifen: targets E2F3/SOX2/Wnt pathways; induces apoptosis; inhibits proliferation and migration.	Inhibits broad immune cell functions; induces lymphocyte apoptosis; may systemically suppress immune responses.	May induce resistance to immunotherapy and chemotherapy. Clinical application (as adjuvant), though protumorigenic risks reported.	(263–269)
Simvastatin	Murine Colon Cancer (C26)	Liposome-encapsulated; enhances anticancer effect of 5-fluorouracil.		Preclinical studies (*in vivo*).	(270)
TAPI-1	ESCC*	Metalloproteinase inhibitor; inhibits NF-κB pathway to reduce inflammatory signaling; suppresses tumor growth, migration, and invasion.		Preclinical studies (*in vitro*).	(271)
Trametinib + Palbociclib	Pancreatic Cancer	MEK + CDK4/6 inhibition induces tumor senescence and SASP (containing VEGF), remodeling tumor vasculature.	Promotes CD8^+^ T cell infiltration via VCAM-1, though T cells exhibit exhaustion.	Enhances PD-1 blockade efficacy (Preclinical): Combination therapy converts “cold” tumors to “hot” tumors, significantly improving anti-PD-1 efficacy.	(272)

* ICIs, immune checkpoint inhibitors; CAR-T, chimeric antigen receptor T cell therapy; HNSCC, Head and Neck Squamous Cell Carcinoma; NSCLC, Non-Small Cell Lung Cancer; CRPC, Castration-Resistant Prostate Cancer; CRP, C-reactive protein; TNBC, Triple-Negative Breast Cancer; Melanoma, Melanoma; PDAC, Pancreatic Ductal Adenocarcinoma; AML, Acute Myeloid Leukemia; HGSOC, High-Grade Serous Ovarian Cancer; aCML, Atypical chronic myeloid leukemia; CNL, chronic neutrophilic leukemia; ESCC, Esophageal Squamous Cell Carcinoma; SERM, Selective estrogen receptor modulator.

At the cellular level, aging induces lysosomal dysfunction in activated T cells, which impairs the effective degradation of damaged mitochondria. Instead, these cells release necrotic mitochondria and mitochondrial DNA into the extracellular space via exocytosis, perpetuating the immunosenescence cycle (28). A potential strategy to counter this involves the intercellular transfer of healthy, stem-cell-derived mitochondria to cells with mitochondrial dysfunction, particularly T cells, to restore aerobic respiration, prevent cell death, and recover cellular function (273). Elevated expression of the E3 ubiquitin ligase BFAR in aged individuals inhibits the generation of TRM, thereby suppressing the differentiation of CD8^+^ T cells into the TRM lineage. Targeting this pathway with a small molecule inhibitor of BFAR, iBFAR2, can enhance sensitivity to PD-1 antibody-mediated immunotherapy while mitigating the side effect of systemic inflammation associated with high-dose PD-1 antibodies (31). Inhibiting the formation of PD-L1-containing tEVs, or targeting lipid metabolism or CREB signaling, can reverse T-cell senescence and sensitize tumors to PD-L1 inhibitor therapy (39). Sensitization of aged individuals to immunotherapy can be achieved by targeting the delivery of rejuvenating agents, such as rapamycin, to tumor-draining lymph nodes via a bioorthogonal click chemistry approach, thereby reversing T cell senescence and restoring anti-tumor immunity (274).

## 5 Discussion

Aging remodels the TME through multiple dimensions—fostering an immunosuppressive, pro-fibrotic, and metabolically abnormal niche—which is a fundamental reason for the high incidence, aggressive biology, and therapeutic resistance of cancer in the elderly. However, this does not preclude older cancer patients from deriving benefit from immunotherapy. Across various tumor types, including NSCLC (141, 143–146, 164, 165) and colorectal cancer (158, 167), older cohorts have demonstrated outcomes equivalent or even superior to those of non-older populations, although some results remain contentious due to limitations in sample size and subgroup characteristics. By contrast, elderly patients with cancers like prostate cancer (160, 174) drive less benefit, highlighting marked inter-tumoral heterogeneity in immunotherapy efficacy. Moreover, the poorer tolerance of combination regimens in the elderly population underscores the application and strategic use of immunotherapy in this demographic remain pressing issues requiring resolution (7).

The partial overlap between aging and tumorigenesis provides a rationale for targeting aging-related mechanisms to enhance anticancer immunity (128). For instance, suppressing chronic inflammation via SASP modulation may delay tumor progression, while improving mitochondrial function in aged T cells could restore their antitumor activity (273). Strategies targeting the senescence-associated microenvironment and SASP have demonstrated significant potential in preclinical studies to remodel the tumor immune landscape (177, 211–213). Nevertheless, their clinical translation, particularly for elderly cancer patients, remains constrained by multiple challenges.

Foremost, the complexity and pleiotropic effects of these mechanisms require rigorous clarification. Senescence is a double-edged sword: while SASP promotes tumors in early stages, it may activate immunosurveillance in specific contexts (211, 212). Currently, the effects of most agents, such as rapamycin (178–180) and dexamethasone (263–269), on immune cells are dose- and context-dependent. Consequently, systemic inhibition of SASP or indiscriminate clearance of senescent cells may disrupt normal tissue repair and immune function (210), thereby introducing unpredictable long-term risks.

Second, clinical translation is limited by methodological constraints. Most evidence derives from mouse models or *in vitro* assays (181, 213) that cannot recapitulate the physiological decline, inflammaging, and multimorbidity characteristic of elderly hosts (211, 212). The pharmacokinetic and pharmacodynamic profiles of elderly patients differ significantly from the general population (5); thus, combination therapies, such as senolytics combined with ICIs inhibitors (211, 212, 214) or chemotherapy (213) may exacerbate cumulative toxicity, negatively impacting the performance status and quality of life. In the context of current clinical regimens, such as multi-drug combinations of ICIs and chemotherapy for gastric cancer, the tolerability of elderly patients is a critical factor for consideration in clinical practice.

Furthermore, the severe underrepresentation of elderly population in clinical trials (185–193) introduces inherent bias when extrapolating existing evidence to this demographic. Future research should prioritize patient stratification based on senescence biomarkers, such as SASP factor profiling (210), and the design of adaptive clinical trials specifically tailored for elderly cancer patients that incorporate frailty assessments and polypharmacy management (211–213). Future investigations may fruitfully focus on these areas to elucidate the underlying mechanisms, enabling more targeted immunotherapeutic interventions for older cancer patients to improve efficacy and clinical outcomes. Only through the deep integration of senescence biology mechanisms with geriatric clinical practice can targeting senescence be transformed from a laboratory concept into a precision immunotherapy paradigm that benefits elderly cancer patients.

## Glossary

TME: tumor microenvironment
ICIs: immune checkpoint inhibitors
CAR-T: chimeric antigen receptor T cell therapy
TCR: T-cell receptor
TNF-α: Tumor Necrosis Factor-alpha
IL-6: Interleukin-6
IL-1: Interleukin-1
CRP: C-reactive protein
NK cells: natural killer cells
DCs: dendritic cells
ROS: reactive oxygen species
CXCL4: C-X-C motif chemokine ligand 4
NKG2D: NK group 2 member D
AMPK: AMP-activated protein kinase
SASP: Senescence-Associated Secretory Phenotype
TRM: tissue-resident memory T cells
BFAR: Bifunctional Apoptosis Regulator
ECM: extracellular matrix
CAFs: cancer-associated fibroblasts
tEVs: tumor-derived extracellular vesicles
PD-L1: Programmed death-ligand 1
CREB: cAMP response element-binding protein
PD-1: Programmed cell death protein 1
Tregs: regulatory T cells
JAK: janus kinase
STAT: signal transducer and activator of transcription
cDC1s: conventional type 1 dendritic cells
Th1: Type 1 T helper cells
TAMs: tumor-associated macrophages
TGF-β: Transforming Growth Factor-beta
IL-10: Interleukin-10
EMT: epithelial-mesenchymal transition
iCAFs: inflammatory CAFs
OS: overall survival
PFS: progression-free survival
MMP: matrix metalloproteinases
VEGF: vascular endothelial growth factor
FGF: fibroblast growth factor
bFGF: basic FGF
MDSCs: myeloid-derived suppressor cells
ICAM1: intercellular adhesion molecule 1
HAPLN1: hyaluronan and proteoglycan link protein 1)
NO: nitric oxide
CXCL1: C-X-C motif chemokine ligand 1
CCL8: C-C motif chemokine ligand 8
IL-8: Interleukin-8
TPI: tumor-promoting inflammation
IFN-γ: Interferon-gamma
IL-18: Interleukin-18
IL-1R: IL-1 receptor
IL-17: Interleukin-17
IL-22: Interleukin-22
IL-18BP: IL-18 binding protein
ACSL6: acyl-CoA synthetase 6
ACSL6 pS674: ACSL6 at serine 674
HSC: hematopoietic stem cell
IFN-I: type I interferon
MHC: major histocompatibility complex
HLA: human leukocyte antigen
IL-15: Interleukin-15
Tim-3: T cell immunoglobulin and mucin domain-containing protein 3
NSCLC: non-small cell lung cancer
ORR: objective response rate
mOS: median OS
RCTs: randomized controlled trials
CIMP-high: CpG island methylator phenotype
MSI: microsatellite instability
dMMR: mismatch repair deficiency
mCRC: metastatic colorectal cancer
DCR: disease control rate
CR: complete response
HER2: human epidermal growth factor receptor 2
TNBC: triple-negative breast cancer
pCR: pathological complete response
TILs: tumor-infiltrating lymphocytes
TLS: tertiary lymphoid structures.

## References

[1] Stead ER Bjedov I . Balancing DNA repair to prevent ageing and cancer. Exp Cell Res. (2021) 405:112679. doi: 10.1016/j.yexcr.2021.112679, PMID: 34102225 PMC8361780

[2] Hakim FT Flomerfelt FA Boyiadzis M Gress RE . Aging, immunity and cancer. Curr Opin Immunol. (2004) 16:151–6. doi: 10.1016/j.coi.2004.01.009, PMID: 15023406

[3] Nikolich-Žugich J . The twilight of immunity: emerging concepts in aging of the immune system. Nat Immunol. (2018) 19:10–9. doi: 10.1038/s41590-017-0006-x, PMID: 29242543

[4] Shan T Ran X Li H Feng G Zhang S Zhang X . Disparities in stage at diagnosis for liver cancer in China. J Natl Cancer Cent. (2023) 3:7–13. doi: 10.1016/j.jncc.2022.12.002, PMID: 39036312 PMC11256694

[5] Kadambi S Loh KP Dunne R Magnuson A Maggiore R Zittel J . Older adults with cancer and their caregivers — current landscape and future directions for clinical care. Nat Rev Clin Oncol. (2020) 17:742–55. doi: 10.1038/s41571-020-0421-z, PMID: 32879429 PMC7851836

[6] Wildiers H Heeren P Puts M Topinkova E Janssen-Heijnen MLG Extermann M . International society of geriatric oncology consensus on geriatric assessment in older patients with cancer. J Clin Oncol. (2014) 32:2595–603. doi: 10.1200/JCO.2013.54.8347, PMID: 25071125 PMC4876338

[7] Marosi C Köller M . Challenge of cancer in the elderly. ESMO Open. (2016) 1:e000020. doi: 10.1136/esmoopen-2015-000020, PMID: 27843603 PMC5070391

[8] Mogilenko DA Shchukina I Artyomov MN . Immune ageing at single-cell resolution. Nat Rev Immunol. (2022) 22:484–98. doi: 10.1038/s41577-021-00646-4, PMID: 34815556 PMC8609266

[9] Linton PJ Dorshkind K . Age-related changes in lymphocyte development and function. Nat Immunol. (2004) 5:133–9. doi: 10.1038/ni1033, PMID: 14749784

[10] Frasca D Landin AM Riley RL Blomberg BB . Mechanisms for decreased function of B cells in aged mice and humans. J Immunol. (2008) 180:2741–6. doi: 10.4049/jimmunol.180.5.2741, PMID: 18292491

[11] Goronzy JJ Weyand CM . Understanding immunosenescence to improve responses to vaccines. Nat Immunol. (2013) 14:428–36. doi: 10.1038/ni.2588, PMID: 23598398 PMC4183346

[12] Santoro A Bientinesi E Monti D . Immunosenescence and inflammaging in the aging process: age-related diseases or longevity? Ageing Res Rev. (2021) 71:101422. doi: 10.1016/j.arr.2021.101422, PMID: 34391943

[13] Goronzy JJ Weyand CM . Successful and maladaptive T cell aging. Immunity. (2017) 46:364–78. doi: 10.1016/j.immuni.2017.03.010, PMID: 28329703 PMC5433436

[14] Ferrucci L Fabbri E . Inflammageing: chronic inflammation in ageing, cardiovascular disease, and frailty. Nat Rev Cardiol. (2018) 15:505–22. doi: 10.1038/s41569-018-0064-2, PMID: 30065258 PMC6146930

[15] Franceschi C Campisi J . Chronic inflammation (Inflammaging) and its potential contribution to age-associated diseases. J Gerontol A Biol Sci Med Sci. (2014) 69:S4–9. doi: 10.1093/gerona/glu057, PMID: 24833586

[16] Singh T Newman AB . Inflammatory markers in population studies of aging. Ageing Res Rev. (2011) 10:319–29. doi: 10.1016/j.arr.2010.11.002, PMID: 21145432 PMC3098911

[17] Ling S Xu JW . Phenotypes and functions of “aged” neutrophils in cardiovascular diseases. BioMed Pharmacother. (2024) 179:117324. doi: 10.1016/j.biopha.2024.117324, PMID: 39216451

[18] Chougnet CA Thacker RI Shehata HM Hennies CM Lehn MA Lages CS . Loss of phagocytic and antigen cross-presenting capacity in aging dendritic cells is associated with mitochondrial dysfunction. J Immunol. (2015) 195:2624–32. doi: 10.4049/jimmunol.1501006, PMID: 26246142 PMC4561185

[19] Costantini A Viola N Berretta A Galeazzi R Matacchione G Sabbatinelli J . Age-related M1/M2 phenotype changes in circulating monocytes from healthy/unhealthy individuals. Aging. (2018) 10:1268–80. doi: 10.18632/aging.101465, PMID: 29885276 PMC6046240

[20] Jin WN Shi K He W Sun JH Van Kaer L Shi FD . Neuroblast senescence in the aged brain augments natural killer cell cytotoxicity leading to impaired neurogenesis and cognition. Nat Neurosci. (2021) 24:61–73. doi: 10.1038/s41593-020-00745-w, PMID: 33257875

[21] Qiu Z Li Z Zhang C Zhao Q Liu Z Cheng Q . NK cell senescence in cancer: From molecular mechanisms to therapeutic opportunities. Aging Dis. (2025) 17:1002–33. doi: 10.14336/AD.2025.0053, PMID: 40249925 PMC12834417

[22] Malmberg KJ Carlsten M Björklund A Sohlberg E Bryceson YT Ljunggren HG . Natural killer cell-mediated immunosurveillance of human cancer. Semin Immunol. (2017) 31:20–9. doi: 10.1016/j.smim.2017.08.002, PMID: 28888619

[23] Romero M Miller K Gelsomini A Garcia D Li K Suresh D . Immunometabolic effects of lactate on humoral immunity in healthy individuals of different ages. Nat Commun. (2024) 15:7515. doi: 10.1038/s41467-024-51207-x, PMID: 39209820 PMC11362567

[24] Rousseau L Hajdu KL Ho PC . Meta-epigenetic shifts in T cell aging and aging-related dysfunction. J BioMed Sci. (2025) 32:51. doi: 10.1186/s12929-025-01146-6, PMID: 40410784 PMC12101013

[25] Ferrucci L Corsi A Lauretani F Bandinelli S Bartali B Taub DD . The origins of age-related proinflammatory state. Blood. (2005) 105:2294–9. doi: 10.1182/blood-2004-07-2599, PMID: 15572589 PMC9828256

[26] Wang B Han J Elisseeff JH Demaria M . The senescence-associated secretory phenotype and its physiological and pathological implications. Nat Rev Mol Cell Biol. (2024) 25:958–78. doi: 10.1038/s41580-024-00727-x, PMID: 38654098

[27] Davalos AR Coppe JP Campisi J Desprez PY . Senescent cells as a source of inflammatory factors for tumor progression. Cancer Metastasis Rev. (2010) 29:273–83. doi: 10.1007/s10555-010-9220-9, PMID: 20390322 PMC2865636

[28] Jin J Mu Y Zhang H Sturmlechner I Wang C Jadhav RR . CISH impairs lysosomal function in activated T cells resulting in mitochondrial DNA release and inflammaging. Nat Aging. (2023) 3:600–16. doi: 10.1038/s43587-023-00399-w, PMID: 37118554 PMC10388378

[29] Desdín-Micó G Soto-Heredero G Aranda JF Oller J Carrasco E Gabandé-Rodríguez E . T cells with dysfunctional mitochondria induce multimorbidity and premature senescence. Science. (2020) 368:1371–6. doi: 10.1126/science.aax0860, PMID: 32439659 PMC7616968

[30] Ovadya Y Landsberger T Leins H Vadai E Gal H Biran A . Impaired immune surveillance accelerates accumulation of senescent cells and aging. Nat Commun. (2018) 9:5435. doi: 10.1038/s41467-018-07825-3, PMID: 30575733 PMC6303397

[31] Pei S Deng X Yang R Wang H Shi JH Wang X . Age-related decline in CD8+ tissue resident memory T cells compromises antitumor immunity. Nat Aging. (2024) 4:1828–44. doi: 10.1038/s43587-024-00746-5, PMID: 39592880

[32] Han B Zheng R Zeng H Wang S Sun K Chen R . Cancer incidence and mortality in China, 2022. J Natl Cancer Cent. (2024) 4:47–53. doi: 10.1016/j.jncc.2024.01.006, PMID: 39036382 PMC11256708

[33] López-Otín C Pietrocola F Roiz-Valle D Galluzzi L Kroemer G . Meta-hallmarks of aging and cancer. Cell Metab. (2023) 35:12–35. doi: 10.1016/j.cmet.2022.11.001, PMID: 36599298

[34] Coussens LM Werb Z . Inflammation and cancer. Nature. (2002) 420:860–7. doi: 10.1038/nature01322, PMID: 12490959 PMC2803035

[35] Bhowmick NA Neilson EG Moses HL . Stromal fibroblasts in cancer initiation and progression. Nature. (2004) 432:332–7. doi: 10.1038/nature03096, PMID: 15549095 PMC3050735

[36] Pickup MW Mouw JK Weaver VM . The extracellular matrix modulates the hallmarks of cancer. EMBO Rep. (2014) 15:1243–53. doi: 10.15252/embr.201439246, PMID: 25381661 PMC4264927

[37] Rimal R Desai P Daware R Hosseinnejad A Prakash J Lammers T . Cancer-associated fibroblasts: Origin, function, imaging, and therapeutic targeting. Adv Drug Delivery Rev. (2022) 189:114504. doi: 10.1016/j.addr.2022.114504, PMID: 35998825

[38] Lu P Weaver VM Werb Z . The extracellular matrix: A dynamic niche in cancer progression. J Cell Biol. (2012) 196:395–406. doi: 10.1083/jcb.201102147, PMID: 22351925 PMC3283993

[39] Ma F Liu X Zhang Y Tao Y Zhao L Abusalamah H . Tumor extracellular vesicle–derived PD-L1 promotes T cell senescence through lipid metabolism reprogramming. Sci Transl Med. (2025) 17:eadm7269. doi: 10.1126/scitranslmed.adm7269, PMID: 39937879 PMC12063564

[40] Dragu LD Chivu-Economescu M Pitica IM Matei L Bleotu C Diaconu CC . Targeting exosomal PD-L1 as a new frontier in cancer immunotherapy. Curr Issues Mol Biol. (2025) 47:525. doi: 10.3390/cimb47070525, PMID: 40728994 PMC12293241

[41] Mi B Xiong Y Knoedler S Alfertshofer M Panayi AC Wang H . Ageing-related bone and immunity changes: insights into the complex interplay between the skeleton and the immune system. Bone Res. (2024) 12:42. doi: 10.1038/s41413-024-00346-4, PMID: 39103328 PMC11300832

[42] Chen ACY Jaiswal S Martinez D Yerinde C Ji K Miranda V . The aged tumor microenvironment limits T cell control of cancer. Nat Immunol. (2024) 25:1033–45. doi: 10.1038/s41590-024-01828-7, PMID: 38745085 PMC11500459

[43] Zhivaki D Kennedy SN Park J Boriello F Devant P Cao A . Correction of age-associated defects in dendritic cells enables CD4+ T cells to eradicate tumors. Cell. (2024) 187:3888–3903.e18. doi: 10.1016/j.cell.2024.05.026, PMID: 38870946 PMC11283364

[44] Mantovani A Marchesi F Malesci A Laghi L Allavena P . Tumor-associated macrophages as treatment targets in oncology. Nat Rev Clin Oncol. (2017) 14:399–416. doi: 10.1038/nrclinonc.2016.217, PMID: 28117416 PMC5480600

[45] Wynn TA Vannella KM . Macrophages in tissue repair, regeneration, and fibrosis. Immunity. (2016) 44:450–62. doi: 10.1016/j.immuni.2016.02.015, PMID: 26982353 PMC4794754

[46] Wu L Lin H Li S Huang Y Sun Y Shu S . Macrophage iron dyshomeostasis promotes aging-related renal fibrosis. Aging Cell. (2024) 23:e14275. doi: 10.1111/acel.14275, PMID: 39016438 PMC11561705

[47] Pawelec G . Age and immunity: what is “immunosenescence”? Exp Gerontol. (2018) 105:4–9. doi: 10.1016/j.exger.2017.10.024, PMID: 29111233

[48] Ren LL Miao H Wang YN Liu F Li P Zhao YY . TGF-β as A master regulator of aging-associated tissue fibrosis. Aging Dis. (2023) 14:1633. doi: 10.14336/AD.2023.0222, PMID: 37196129 PMC10529747

[49] Orimo A Gupta PB Sgroi DC Arenzana-Seisdedos F Delaunay T Naeem R . Stromal fibroblasts present in invasive human breast carcinomas promote tumor growth and angiogenesis through elevated SDF-1/CXCL12 secretion. Cell. (2005) 121:335–48. doi: 10.1016/j.cell.2005.02.034, PMID: 15882617

[50] Liu L Liu Z Meng L Li L Gao J Yu S . An integrated fibrosis signature for predicting survival and immunotherapy efficacy of patients with hepatocellular carcinoma. Front Mol Biosci. (2021) 8:766609. doi: 10.3389/fmolb.2021.766609, PMID: 34970594 PMC8712696

[51] Fan JQ Wang MF Chen HL Shang D Das JK Song J . Current advances and outlooks in immunotherapy for pancreatic ductal adenocarcinoma. Mol Cancer. (2020) 19:32. doi: 10.1186/s12943-020-01151-3, PMID: 32061257 PMC7023714

[52] Ma C Yang C Peng A Sun T Ji X Mi J . Pan-cancer spatially resolved single-cell analysis reveals the crosstalk between cancer-associated fibroblasts and tumor microenvironment. Mol Cancer. (2023) 22:170. doi: 10.1186/s12943-023-01876-x, PMID: 37833788 PMC10571470

[53] Wang H Yu Y Li R Zhang H Chen ZS Sun C . Immunoregulatory mechanisms in the aging microenvironment: Targeting the senescence-associated secretory phenotype for cancer immunotherapy. Acta Pharm Sin B. (2025) 15:4476–96. doi: 10.1016/j.apsb.2025.07.022, PMID: 41049742 PMC12491696

[54] Whitehead M Antonazzi M Shanahan CM . Extracellular vesicles: The key to unlocking mechanisms of age-related vascular disease? J Cardiovasc Aging. (2024) 4:12. Available online at: https://www.oaepublish.com/articles/jca.2023.49 (Accessed March 2, 2026).

[55] Glaviano A Lau HSH Carter LM Lee EHC Lam HY Okina E . Harnessing the tumor microenvironment: Targeted cancer therapies through modulation of epithelial-mesenchymal transition. J Hematol OncolJ Hematol Oncol. (2025) 18:6. doi: 10.1186/s13045-024-01634-6, PMID: 39806516 PMC11733683

[56] Marino-Bravante GE Carey AE Hüser L Dixit A Wang V Kaur A . Age-dependent loss of HAPLN1 erodes vascular integrity via indirect upregulation of endothelial ICAM1 in melanoma. Nat Aging. (2024) 4:350–63. doi: 10.1038/s43587-024-00581-8, PMID: 38472454 PMC13242075

[57] Schwab N Leung E Hazrati LN . Cellular senescence in traumatic brain injury: Evidence and perspectives. Front Aging Neurosci. (2021) 13:742632. doi: 10.3389/fnagi.2021.742632, PMID: 34650425 PMC8505896

[58] Hoechst B Gamrekelashvili J Manns MP Greten TF Korangy F . Plasticity of human Th17 cells and iTregs is orchestrated by different subsets of myeloid cells. Blood. (2011) 117:6532–41. doi: 10.1182/blood-2010-11-317321, PMID: 21493801

[59] Hu T Zhai J Yang Z Peng J Wang C Liu X . Myeloid-derived suppressor cells in cancer: Mechanistic insights and targeted therapeutic innovations. MedComm. (2025) 6:e70231. doi: 10.1002/mco2.70231, PMID: 40452814 PMC12126600

[60] Hanahan D Weinberg RA . Hallmarks of cancer: the next generation. Cell. (2011) 144:646–74. doi: 10.1016/j.cell.2011.02.013, PMID: 21376230

[61] Biragyn A Ferrucci L . Gut dysbiosis: a potential link between increased cancer risk in ageing and inflammaging. Lancet Oncol. (2018) 19:e295–304. doi: 10.1016/S1470-2045(18)30095-0, PMID: 29893261 PMC6047065

[62] Park MD Le Berichel J Hamon P Wilk CM Belabed M Yatim N . Hematopoietic aging promotes cancer by fueling IL-1α–driven emergency myelopoiesis. Science. (2024) 386:eadn0327. doi: 10.1126/science.adn0327, PMID: 39236155 PMC7616710

[63] Mantovani A Allavena P Sica A Balkwill F . Cancer-related inflammation. Nature. (2008) 454:436–44. doi: 10.1038/nature07205, PMID: 18650914

[64] Kureshi CT Dougan SK . Cytokines in cancer. Cancer Cell. (2025) 43:15–35. doi: 10.1016/j.ccell.2024.11.011, PMID: 39672170 PMC11841838

[65] López-Otín C Blasco MA Partridge L Serrano M Kroemer G . Hallmarks of aging: An expanding universe. Cell. (2023) 186:243–78. doi: 10.1016/j.cell.2022.11.001, PMID: 36599349

[66] Orange ST Leslie J Ross M Mann DA Wackerhage H . The exercise IL-6 enigma in cancer. Trends Endocrinol Metab. (2023) 34:749–63. doi: 10.1016/j.tem.2023.08.001, PMID: 37633799

[67] Heikkilä K Ebrahim S Lawlor DA . Systematic review of the association between circulating interleukin-6 (IL-6) and cancer. Eur J Cancer. (2008) 44:937–45. doi: 10.1016/j.ejca.2008.02.047, PMID: 18387296

[68] Johnson DE O’Keefe RA Grandis JR . Targeting the IL-6/JAK/STAT3 signaling axis in cancer. Nat Rev Clin Oncol. (2018) 15:234–48. doi: 10.1038/nrclinonc.2018.8, PMID: 29405201 PMC5858971

[69] Buechler MB Pradhan RN Krishnamurty AT Cox C Calviello AK Wang AW . Cross-tissue organization of the fibroblast lineage. Nature. (2021) 593:575–9. doi: 10.1038/s41586-021-03549-5, PMID: 33981032

[70] Ershler WB Sun WH Binkley N Gravenstein S Volk MJ Kamoske G . Interleukin-6 and aging: blood levels and mononuclear cell production increase with advancing age and *in vitro* production is modifiable by dietary restriction. Lymphokine Cytokine Res. (1993) 12:225–30. 8218595

[71] Fujimoto M Nakano M Terabe F Kawahata H Ohkawara T Han Y . The influence of excessive IL-6 production *in vivo* on the development and function of foxp3+ Regulatory T cells. J Immunol. (2011) 186:32–40. doi: 10.4049/jimmunol.0903314, PMID: 21106853

[72] Xie TX Wei D Liu M Gao AC Ali-Osman F Sawaya R . Stat3 activation regulates the expression of matrix metalloproteinase-2 and tumor invasion and metastasis. Oncogene. (2004) 23:3550–60. doi: 10.1038/sj.onc.1207383, PMID: 15116091

[73] Wei LH Kuo ML Chen CA Chou CH Lai KB Lee CN . Interleukin-6 promotes cervical tumor growth by VEGF-dependent angiogenesis via a STAT3 pathway. Oncogene. (2003) 22:1517–27. doi: 10.1038/sj.onc.1206226, PMID: 12629515

[74] Hodge DR Peng B Cherry JC Hurt EM Fox SD Kelley JA . Interleukin 6 supports the maintenance of p53 tumor suppressor gene promoter methylation. Cancer Res. (2005) 65:4673–82. doi: 10.1158/0008-5472.CAN-04-3589, PMID: 15930285

[75] Kumari N Dwarakanath BS Das A Bhatt AN . Role of interleukin-6 in cancer progression and therapeutic resistance. Tumor Biol. (2016) 37:11553–72. doi: 10.1007/s13277-016-5098-7, PMID: 27260630

[76] Mantovani A Dinarello CA Molgora M Garlanda C . Interleukin-1 and related cytokines in the regulation of inflammation and immunity. Immunity. (2019) 50:778–95. doi: 10.1016/j.immuni.2019.03.012, PMID: 30995499 PMC7174020

[77] Lamkanfi M Dixit VM . Mechanisms and functions of inflammasomes. Cell. (2014) 157:1013–22. doi: 10.1016/j.cell.2014.04.007, PMID: 24855941

[78] Dmitrieva-Posocco O Dzutsev A Posocco DF Hou V Yuan W Thovarai V . Cell-type-specific responses to interleukin-1 control microbial invasion and tumor-elicited inflammation in colorectal cancer. Immunity. (2019) 50:166–180.e7. doi: 10.1016/j.immuni.2018.11.015, PMID: 30650375 PMC6490968

[79] Alinejad V Dolati S Motallebnezhad M Yousefi M . The role of IL17B-IL17RB signaling pathway in breast cancer. BioMed Pharmacother. (2017) 88:795–803. doi: 10.1016/j.biopha.2017.01.120, PMID: 28160754

[80] Sinha VC Rinkenbaugh AL Xu M Zhou X Zhang X Jeter-Jones S . Single-cell evaluation reveals shifts in the tumor-immune niches that shape and maintain aggressive lesions in the breast. Nat Commun. (2021) 12:5024. doi: 10.1038/s41467-021-25240-z, PMID: 34408137 PMC8373912

[81] Ma HY Yamamoto G Xu J Liu X Karin D Kim JY . IL-17 signaling in steatotic hepatocytes and macrophages promotes hepatocellular carcinoma in alcohol-related liver disease. J Hepatol. (2020) 72:946–59. doi: 10.1016/j.jhep.2019.12.016, PMID: 31899206 PMC7167339

[82] Numasaki M Watanabe M Suzuki T Takahashi H Nakamura A McAllister F . IL-17 enhances the net angiogenic activity and *in vivo* growth of human non-small cell lung cancer in SCID mice through promoting CXCR-2-dependent angiogenesis. J Immunol. (2005) 175:6177–89. doi: 10.4049/jimmunol.175.9.6177, PMID: 16237115

[83] Chang SH Mirabolfathinejad SG Katta H Cumpian AM Gong L Caetano MS . T helper 17 cells play a critical pathogenic role in lung cancer. Proc Natl Acad Sci. (2014) 111:5664–9. doi: 10.1073/pnas.1319051111, PMID: 24706787 PMC3992670

[84] Wu S Rhee KJ Albesiano E Rabizadeh S Wu X Yen HR . A human colonic commensal promotes colon tumorigenesis via activation of T helper type 17 T cell responses. Nat Med. (2009) 15:1016–22. doi: 10.1038/nm.2015, PMID: 19701202 PMC3034219

[85] Zhang Y Zoltan M Riquelme E Xu H Sahin I Castro-Pando S . Immune cell production of interleukin 17 induces stem cell features of pancreatic intraepithelial neoplasia cells. Gastroenterology. (2018) 155:210–223.e3. doi: 10.1053/j.gastro.2018.03.041, PMID: 29604293 PMC6035075

[86] McAllister F Bailey JM Alsina J Nirschl CJ Sharma R Fan H . Oncogenic kras activates a hematopoietic-to-epithelial IL-17 signaling axis in preinvasive pancreatic neoplasia. Cancer Cell. (2014) 25:621–37. doi: 10.1016/j.ccr.2014.03.014, PMID: 24823639 PMC4072043

[87] Loncle C Bonjoch L Folch-Puy E Lopez-Millan MB Lac S Molejon MI . IL17 functions through the novel REG3β–JAK2–STAT3 inflammatory pathway to promote the transition from chronic pancreatitis to pancreatic cancer. Cancer Res. (2015) 75:4852–62. doi: 10.1158/0008-5472.CAN-15-0896, PMID: 26404002 PMC4651828

[88] Dudakov JA Hanash AM Van Den Brink MRM . Interleukin-22: immunobiology and pathology. Annu Rev Immunol. (2015) 33:747–85. doi: 10.1146/annurev-immunol-032414-112123, PMID: 25706098 PMC4407497

[89] Yasuda K Nakanishi K Tsutsui H . Interleukin-18 in health and disease. Int J Mol Sci. (2019) 20:649. doi: 10.3390/ijms20030649, PMID: 30717382 PMC6387150

[90] Shen J Zhang Y Tang W Yang M Cheng T Chen Y . Short IL-18 generated by caspase-3 cleavage mobilizes NK cells to suppress tumor growth. Nat Immunol. (2025) 26:416–28. doi: 10.1038/s41590-024-02074-7, PMID: 39891018

[91] Zhou T Damsky W Weizman OE McGeary MK Hartmann KP Rosen CE . IL-18BP is a secreted immune checkpoint and barrier to IL-18 immunotherapy. Nature. (2020) 583:609–14. doi: 10.1038/s41586-020-2422-6, PMID: 32581358 PMC7381364

[92] Di Y Wang Z Xiao J Zhang X Ye L Wen X . ACSL6-activated IL-18R1–NF-κB promotes IL-18–mediated tumor immune evasion and tumor progression. Sci Adv. (2024) 10:eadp0719. doi: 10.1126/sciadv.adp0719, PMID: 39292786 PMC11409972

[93] Molgora M Bonavita E Ponzetta A Riva F Barbagallo M Jaillon S . IL-1R8 is a checkpoint in NK cells regulating anti-tumor and anti-viral activity. Nature. (2017) 551:110–4. doi: 10.1038/nature24293, PMID: 29072292 PMC5768243

[94] Salminen A . Activation of immunosuppressive network in the aging process. Ageing Res Rev. (2020) 57:100998. doi: 10.1016/j.arr.2019.100998, PMID: 31838128

[95] Hoekstra ME Slagter M Urbanus J Toebes M Slingerland N De Rink I . Distinct spatiotemporal dynamics of CD8+ T cell-derived cytokines in the tumor microenvironment. Cancer Cell. (2024) 42:157–167.e9. doi: 10.1016/j.ccell.2023.12.010, PMID: 38194914 PMC10783802

[96] Zelová H Hošek J . TNF-α signaling and inflammation: Interactions between old acquaintances. Inflammation Res. (2013) 62:641–51. doi: 10.1007/s00011-013-0633-0, PMID: 23685857

[97] Verhoef C De Wilt JHW Grünhagen DJ Van Geel AN Ten Hagen TLM Eggermont AMM . Isolated limb perfusion with melphalan and TNF-α in the treatment of extremity sarcoma. Curr Treat Option Oncol. (2007) 8:417–27. doi: 10.1007/s11864-007-0044-y, PMID: 18066703 PMC2781100

[98] Lin X Zheng W Liu J Zhang Y Qin H Wu H . Oxidative stress in Malignant melanoma enhances tumor necrosis factor-α secretion of tumor-associated macrophages that promote cancer cell invasion. Antioxid Redox Signal. (2013) 19:1337–55. doi: 10.1089/ars.2012.4617, PMID: 23373752

[99] Wang X Yang L Huang F Zhang Q Liu S Ma L . Inflammatory cytokines IL-17 and TNF-α up-regulate PD-L1 expression in human prostate and colon cancer cells. Immunol Lett. (2017) 184:7–14. doi: 10.1016/j.imlet.2017.02.006, PMID: 28223102 PMC5362328

[100] Li CW Xia W Huo L Lim SO Wu Y Hsu JL . Epithelial–mesenchymal transition induced by TNF-α requires NF-κB–mediated transcriptional upregulation of Twist1. Cancer Res. (2012) 72:1290–300. doi: 10.1158/0008-5472.CAN-11-3123, PMID: 22253230 PMC3350107

[101] Bertrand F Rochotte J Colacios C Montfort A Tilkin-Mariamé AF Touriol C . Blocking tumor necrosis factor α enhances CD8 T-cell–dependent immunity in experimental melanoma. Cancer Res. (2015) 75:2619–28. doi: 10.1158/0008-5472.CAN-14-2524, PMID: 25977337

[102] Stewart CM Siegler EL Kenderian SS . The pleiotropic roles of cytokines in chimeric antigen receptor T-cell therapy. Cancer Immunol Res. (2026) 14:10–21. doi: 10.1158/2326-6066.CIR-25-0631, PMID: 41324276 PMC12671922

[103] Chen X Chen F Jia S Lu Q Zhao M . Antigen-presenting fibroblasts: Emerging players in immune modulation and therapeutic targets. Theranostics. (2025) 15:3332–44. doi: 10.7150/thno.104900, PMID: 40093895 PMC11905139

[104] Burnet FM . The concept of immunological surveillance. In: Schwartz RS , editor. Progress in tumor research. Basel, Switzerland: Karger Publishers (1970). p. 1–27. Available online at: https://karger.com/books/book/483/chapter/5575692 (Accessed March 3, 2026). 10.1159/000386035 4921480

[105] Monach PA Meredith SC T.Siegel C Schreiber H . A unique tumor antigen produced by a single amino acid substitution. Immunity. (1995) 2:45–59. doi: 10.1016/1074-7613(95)90078-0, PMID: 7600302

[106] Dunn GP Bruce AT Ikeda H Old LJ Schreiber RD . Cancer immunoediting: from immunosurveillance to tumor escape. Nat Immunol. (2002) 3:991–8. doi: 10.1038/ni1102-991, PMID: 12407406

[107] Tilstra JS Robinson AR Wang J Gregg SQ Clauson CL Reay DP . NF-κB inhibition delays DNA damage–induced senescence and aging in mice. J Clin Invest. (2012) 122:2601–12. doi: 10.1172/JCI45785, PMID: 22706308 PMC3386805

[108] Aman Y Schmauck-Medina T Hansen M Morimoto RI Simon AK Bjedov I . Autophagy in healthy aging and disease. Nat Aging. (2021) 1:634–50. doi: 10.1038/s43587-021-00098-4, PMID: 34901876 PMC8659158

[109] Zhang Y Nicholatos J Dreier JR Ricoult SJH Widenmaier SB Hotamisligil GS . Coordinated regulation of protein synthesis and degradation by mTORC1. Nature. (2014) 513:440–3. doi: 10.1038/nature13492, PMID: 25043031 PMC4402229

[110] Kim J Kundu M Viollet B Guan KL . AMPK and mTOR regulate autophagy through direct phosphorylation of Ulk1. Nat Cell Biol. (2011) 13:132–41. doi: 10.1038/ncb2152, PMID: 21258367 PMC3987946

[111] Villarino AV Kanno Y Ferdinand JR O’Shea JJ . Mechanisms of jak/STAT signaling in immunity and disease. J Immunol. (2014) 194:21–7. doi: 10.4049/jimmunol.1401867, PMID: 25527793 PMC4524500

[112] Xu M Tchkonia T Kirkland JL . Perspective: Targeting the JAK/STAT pathway to fight age-related dysfunction. Pharmacol Res. (2016) 111:152–4. doi: 10.1016/j.phrs.2016.05.015, PMID: 27241018 PMC5026572

[113] Schmitz CRR Maurmann RM Guma FTCR Bauer ME Barbé-Tuana FM . cGAS-STING pathway as a potential trigger of immunosenescence and inflammaging. Front Immunol. (2023) 14:1132653. doi: 10.3389/fimmu.2023.1132653, PMID: 36926349 PMC10011111

[114] Zhou J Zhuang Z Li J Feng Z . Significance of the cGAS-STING pathway in health and disease. Int J Mol Sci. (2023) 24:13316. doi: 10.3390/ijms241713316, PMID: 37686127 PMC10487967

[115] Glück S Ablasser A . Innate immunosensing of DNA in cellular senescence. Curr Opin Immunol. (2019) 56:31–6. doi: 10.1016/j.coi.2018.09.013, PMID: 30296662

[116] Ge Y Zhou M Chen C Wu X Wang X . Role of AMPK mediated pathways in autophagy and aging. Biochimie. (2022) 195:100–13. doi: 10.1016/j.biochi.2021.11.008, PMID: 34838647

[117] Salminen A Kauppinen A Kaarniranta K . AMPK activation inhibits the functions of myeloid-derived suppressor cells (MDSC): Impact on cancer and aging. J Mol Med. (2019) 97:1049–64. doi: 10.1007/s00109-019-01795-9, PMID: 31129755 PMC6647228

[118] Rolf J Zarrouk M Finlay DK Foretz M Viollet B Cantrell DA . AMPKα1: a glucose sensor that controls CD8 T-cell memory. Eur J Immunol. (2013) 43:889–96. doi: 10.1002/eji.201243008, PMID: 23310952 PMC3734624

[119] Acuña-Castroviejo D Escames G Venegas C Díaz-Casado ME Lima-Cabello E López LC . Extrapineal melatonin: Sources, regulation, and potential functions. Cell Mol Life Sci. (2014) 71:2997–3025. doi: 10.1007/s00018-014-1579-2, PMID: 24554058 PMC11113552

[120] Hardeland R . Aging, melatonin, and the pro- and anti-inflammatory networks. Int J Mol Sci. (2019) 20:1223. doi: 10.3390/ijms20051223, PMID: 30862067 PMC6429360

[121] Tang Y Zhou Y Wang Y He Y Ding J Li Y . Ginsenoside Rg1 protects against sca−1+ HSC/HPC cell aging by regulating the SIRT1−FOXO3 and SIRT3−SOD2 signaling pathways in a γ−ray irradiation−induced aging mice model. Exp Ther Med. (2020) 20:1245–52. doi: 10.3892/etm.2020.8810, PMID: 32765665 PMC7388550

[122] Liu TF Yoza BK El Gazzar M Vachharajani VT McCall CE . NAD+-dependent SIRT1 deacetylase participates in epigenetic reprogramming during endotoxin tolerance. J Biol Chem. (2011) 286:9856–64. doi: 10.1074/jbc.M110.196790, PMID: 21245135 PMC3058977

[123] Zhang J Lee SM Shannon S Gao B Chen W Chen A . The type III histone deacetylase Sirt1 is essential for maintenance of T cell tolerance in mice. J Clin Invest. (2009) 119:3048–58. doi: 10.1172/JCI38902, PMID: 19729833 PMC2752073

[124] Bakrim S Fessikh ME Elhrech H Omari NE Amanullah M Ming LC . Targeting inflammation in cancer therapy: From mechanistic insights to emerging therapeutic approaches. J Transl Med. (2025) 23:588. doi: 10.1186/s12967-025-06583-3, PMID: 40420174 PMC12107871

[125] Fu B Meng W Zhao H Zhang B Tang H Zou Y . GRAM domain-containing protein 1A (GRAMD1A) promotes the expansion of hepatocellular carcinoma stem cell and hepatocellular carcinoma growth through STAT5. Sci Rep. (2016) 6:31963. doi: 10.1038/srep31963, PMID: 27585821 PMC5009375

[126] Muroski ME Roycik MD Newcomer RG Van Den Steen PE Opdenakker G Monroe HR . Matrix metalloproteinase-9/gelatinase B is a putative therapeutic target of chronic obstructive pulmonary disease and multiple sclerosis. Curr Pharm Biotechnol. (2008) 9:34–46. doi: 10.2174/138920108783497631, PMID: 18289055

[127] Klein G Vellenga E Fraaije MW Kamps WA De Bont ESJM . The possible role of matrix metalloproteinase (MMP)-2 and MMP-9 in cancer, e.g. acute leukemia. Crit Rev Oncol Hematol. (2004) 50:87–100. doi: 10.1016/j.critrevonc.2003.09.001, PMID: 15157658

[128] McHugh D Durán I Gil J . Senescence as a therapeutic target in cancer and age-related diseases. Nat Rev Drug Discov. (2025) 24:57–71. doi: 10.1038/s41573-024-01074-4, PMID: 39548312

[129] Liu X Mo W Ye J Li L Zhang Y Hsueh EC . Regulatory T cells trigger effector T cell DNA damage and senescence caused by metabolic competition. Nat Commun. (2018) 9:249. doi: 10.1038/s41467-017-02689-5, PMID: 29339767 PMC5770447

[130] Salminen A Kauppinen A Kaarniranta K . Myeloid-derived suppressor cells (MDSC): An important partner in cellular/tissue senescence. Biogerontology. (2018) 19:325–39. doi: 10.1007/s10522-018-9762-8, PMID: 29959657

[131] Salminen A . Feed-forward regulation between cellular senescence and immunosuppression promotes the aging process and age-related diseases. Ageing Res Rev. (2021) 67:101280. doi: 10.1016/j.arr.2021.101280, PMID: 33581314

[132] Ruhland MK Loza AJ Capietto AH Luo X Knolhoff BL Flanagan KC . Stromal senescence establishes an immunosuppressive microenvironment that drives tumorigenesis. Nat Commun. (2016) 7:11762. doi: 10.1038/ncomms11762, PMID: 27272654 PMC4899869

[133] Crespo J Sun H Welling TH Tian Z Zou W . T cell anergy, exhaustion, senescence, and stemness in the tumor microenvironment. Curr Opin Immunol. (2013) 25:214–21. doi: 10.1016/j.coi.2012.12.003, PMID: 23298609 PMC3636159

[134] Lian J Yue Y Yu W Zhang Y . Immunosenescence: A key player in cancer development. J Hematol OncolJ Hematol Oncol. (2020) 13:151. doi: 10.1186/s13045-020-00986-z, PMID: 33168037 PMC7653700

[135] Floess S Freyer J Siewert C Baron U Olek S Polansky J . Epigenetic control of the foxp3 locus in regulatory T cells. PloS Biol. (2007) 5:e38. doi: 10.1371/journal.pbio.0050038, PMID: 17298177 PMC1783672

[136] Guo Z Tilburgs T Wong B Strominger JL . Dysfunction of dendritic cells in aged C57BL/6 mice leads to failure of natural killer cell activation and of tumor eradication. Proc Natl Acad Sci. (2014) 111:14199–204. doi: 10.1073/pnas.1414780111, PMID: 25225399 PMC4191753

[137] Zhao Y Shao Q Peng G . Exhaustion and senescence: Two crucial dysfunctional states of T cells in the tumor microenvironment. Cell Mol Immunol. (2020) 17:27–35. doi: 10.1038/s41423-019-0344-8, PMID: 31853000 PMC6952436

[138] Nokihara H Kijima T Yokoyama T Kagamu H Suzuki T Mori M . Real-World Treatments and Clinical Outcomes in Advanced NSCLC without Actionable Mutations after Introduction of Immunotherapy in Japan. Cancers. (2022) 14:2846. doi: 10.3390/cancers14122846, PMID: 35740512 PMC9220782

[139] Lichtenstein MRL Nipp RD Muzikansky A Goodwin K Anderson D Newcomb RA . Impact of age on outcomes with immunotherapy in patients with non–small cell lung cancer. J Thorac Oncol. (2019) 14:547–52. doi: 10.1016/j.jtho.2018.11.011, PMID: 30476576

[140] Voruganti T Soulos PR Mamtani R Presley CJ Gross CP . Association between age and survival trends in advanced non–small cell lung cancer after adoption of immunotherapy. JAMA Oncol. (2023) 9:334. doi: 10.1001/jamaoncol.2022.6901, PMID: 36701150 PMC9880865

[141] Herbst RS Baas P Kim DW Felip E Pérez-Gracia JL Han JY . Pembrolizumab versus docetaxel for previously treated, PD-L1-positive, advanced non-small-cell lung cancer (KEYNOTE-010): a randomized controlled trial. Lancet. (2016) 387:1540–50. doi: 10.1016/S0140-6736(15)01281-7, PMID: 26712084

[142] Masuda T Fujitaka K Suzuki T Hamai K Matsumoto N Matsumura M . Phase 2 study of first-line pembrolizumab monotherapy in elderly patients with non-small-cell lung cancer expressing high PD-L1. Thorac Cancer. (2022) 13:1611–8. doi: 10.1111/1759-7714.14428, PMID: 35488720 PMC9161325

[143] Mok TSK Wu YL Kudaba I Kowalski DM Cho BC Turna HZ . Pembrolizumab versus chemotherapy for previously untreated, PD-L1-expressing, locally advanced or metastatic non-small-cell lung cancer (KEYNOTE-042): a randomized, open-label, controlled, phase 3 trial. Lancet. (2019) 393:1819–30. doi: 10.1016/S0140-6736(18)32409-7, PMID: 30955977

[144] Gogishvili M Melkadze T Makharadze T Giorgadze D Dvorkin M Penkov K . Cemiplimab plus chemotherapy versus chemotherapy alone in non-small cell lung cancer: a randomized, controlled, double-blind phase 3 trial. Nat Med. (2022) 28:2374–80. doi: 10.1038/s41591-022-01977-y, PMID: 36008722 PMC9671806

[145] Rittmeyer A Barlesi F Waterkamp D Park K Ciardiello F Von Pawel J . Atezolizumab versus docetaxel in patients with previously treated non-small-cell lung cancer (OAK): a phase 3, open-label, multicenter randomized controlled trial. Lancet. (2017) 389:255–65. doi: 10.1016/S0140-6736(16)32517-X, PMID: 27979383 PMC6886121

[146] Socinski MA Nishio M Jotte RM Cappuzzo F Orlandi F Stroyakovskiy D . IMpower150 final overall survival analyses for atezolizumab plus bevacizumab and chemotherapy in first-line metastatic nonsquamous NSCLC. J Thorac Oncol. (2021) 16:1909–24. doi: 10.1016/j.jtho.2021.07.009, PMID: 34311108

[147] Nosaki K Saka H Hosomi Y Baas P De Castro G Reck M . Safety and efficacy of pembrolizumab monotherapy in elderly patients with PD-L1–positive advanced non–small-cell lung cancer: Pooled analysis from the KEYNOTE-010, KEYNOTE-024, and KEYNOTE-042 studies. Lung Cancer. (2019) 135:188–95. doi: 10.1016/j.lungcan.2019.07.004, PMID: 31446994

[148] Shiono A Imai H Wasamoto S Tsuda T Nagai Y Minemura H . Real-world data of atezolizumab plus carboplatin and etoposide in elderly patients with extensive-disease small-cell lung cancer. Cancer Med. (2023) 12:73–83. doi: 10.1002/cam4.4938, PMID: 35699088 PMC9844637

[149] Kang YK Chen LT Ryu MH Oh DY Oh SC Chung HC . Nivolumab plus chemotherapy versus placebo plus chemotherapy in patients with HER2-negative, untreated, unresectable advanced or recurrent gastric or gastro-esophageal junction cancer (ATTRACTION-4): a randomized, multicenter, double-blind, placebo-controlled, phase 3 trial. Lancet Oncol. (2022) 23:234–47. doi: 10.1016/S1470-2045(21)00692-6, PMID: 35030335

[150] Janjigian YY Shitara K Moehler M Garrido M Salman P Shen L . First-line nivolumab plus chemotherapy versus chemotherapy alone for advanced gastric, gastro-esophageal junction, and esophageal adenocarcinoma (CheckMate 649): a randomized, open-label, phase 3 trial. Lancet. (2021) 398:27–40. doi: 10.1016/S0140-6736(21)00797-2, PMID: 34102137 PMC8436782

[151] Kang YK Boku N Satoh T Ryu MH Chao Y Kato K . Nivolumab in patients with advanced gastric or gastro-esophageal junction cancer refractory to, or intolerant of, at least two previous chemotherapy regimens (ONO-4538-12, ATTRACTION-2): a randomized, double-blind, placebo-controlled, phase 3 trial. Lancet. (2017) 390:2461–71. doi: 10.1016/S0140-6736(17)31827-5, PMID: 28993052

[152] Schmid P Cortes J Pusztai L McArthur H Kümmel S Bergh J . Pembrolizumab for early triple-negative breast cancer. N Engl J Med. (2020) 382:810–21. doi: 10.1056/NEJMoa1910549, PMID: 32101663

[153] Li D Toh HC Merle P Tsuchiya K Hernandez S Verret W . Atezolizumab plus Bevacizumab versus Sorafenib for Unresectable Hepatocellular Carcinoma: Results from Older Adults Enrolled in the IMbrave150 Randomized Clinical Trial. Liver Cancer. (2022) 11:558–71. doi: 10.1159/000525671, PMID: 36589722 PMC9801180

[154] Rini BI Plimack ER Stus V Gafanov R Hawkins R Nosov D . Pembrolizumab plus Axitinib versus Sunitinib for Advanced Renal-Cell Carcinoma. N Engl J Med. (2019) 380:1116–27. doi: 10.1056/NEJMoa1816714, PMID: 30779529

[155] Ibrahim T Mateus C Baz M Robert C . Older melanoma patients aged 75 and above retain responsiveness to anti-PD1 therapy: results of a retrospective single-institution cohort study. Cancer Immunol Immunother. (2018) 67:1571–8. doi: 10.1007/s00262-018-2219-8, PMID: 30056599 PMC11028036

[156] Kugel CH Douglass SM Webster MR Kaur A Liu Q Yin X . Age correlates with response to anti-PD1, reflecting age-related differences in intratumoral effector and regulatory T-cell populations. Clin Cancer Res. (2018) 24:5347–56. doi: 10.1158/1078-0432.CCR-18-1116, PMID: 29898988 PMC6324578

[157] Johnpulle RAN Conry RM Sosman JA Puzanov I Johnson DB . Responses to immune checkpoint inhibitors in nonagenarians. OncoImmunology. (2016) 5:e1234572. doi: 10.1080/2162402X.2016.1234572, PMID: 27999751 PMC5139628

[158] Diaz LA Shiu KK Kim TW Jensen BV Jensen LH Punt C . Pembrolizumab versus chemotherapy for microsatellite instability-high or mismatch repair-deficient metastatic colorectal cancer (KEYNOTE-177): final analysis of a randomized, open-label, phase 3 study. Lancet Oncol. (2022) 23:659–70. doi: 10.1016/S1470-2045(22)00197-8, PMID: 35427471 PMC9533375

[159] Saberzadeh-Ardestani B Jones JC Hubbard JM McWilliams RR Halfdanarson TR Shi Q . Association between survival and metastatic site in mismatch repair–deficient metastatic colorectal cancer treated with first-line pembrolizumab. JAMA Netw Open. (2023) 6:e230400. doi: 10.1001/jamanetworkopen.2023.0400, PMID: 36811859 PMC9947726

[160] Petrylak DP Ratta R Matsubara N Korbenfeld E Gafanov R Mourey L . Pembrolizumab plus docetaxel versus docetaxel for previously treated metastatic castration-resistant prostate cancer: the randomized, double-blind, phase III KEYNOTE-921 trial. J Clin Oncol. (2025) 43:1638–49. doi: 10.1200/JCO-24-01283, PMID: 40043230 PMC12058370

[161] Huang XZ Gao P Song Y Sun J Chen XW Zhao JH . Efficacy of immune checkpoint inhibitors and age in cancer patients. Immunotherapy. (2020) 12:587–603. doi: 10.2217/imt-2019-0124, PMID: 32378444

[162] Kasherman L Siu DHW Lee KWC Lord S Marschner I Lewis CR . Efficacy of immune checkpoint inhibitors in older adults with advanced stage cancers: A meta-analysis. J Geriatr Oncol. (2020) 11:508–14. doi: 10.1016/j.jgo.2019.05.013, PMID: 31129081

[163] Corbaux P Maillet D Boespflug A Locatelli-Sanchez M Perier-Muzet M Duruisseaux M . Older and younger patients treated with immune checkpoint inhibitors have similar outcomes in real-life setting. Eur J Cancer. (2019) 121:192–201. doi: 10.1016/j.ejca.2019.08.027, PMID: 31590080

[164] Tagliamento M Frelaut M Baldini C Naigeon M Nencioni A Chaput N . The use of immunotherapy in older patients with advanced non-small cell lung cancer. Cancer Treat Rev. (2022) 106:102394. doi: 10.1016/j.ctrv.2022.102394, PMID: 35472632

[165] Garon EB Rizvi NA Hui R Leighl N Balmanoukian AS Eder JP . Pembrolizumab for the treatment of non–small-cell lung cancer. N Engl J Med. (2015) 372:2018–28. doi: 10.1056/NEJMoa1501824, PMID: 25891174

[166] Van Herck Y Feyaerts A Alibhai S Papamichael D Decoster L Lambrechts Y . Is cancer biology different in older patients? Lancet Health Longev. (2021) 2:e663–77. doi: 10.1016/S2666-7568(21)00179-3, PMID: 36098020

[167] André T Shiu KK Kim TW Jensen BV Jensen LH Punt C . Pembrolizumab in microsatellite-instability–high advanced colorectal cancer. N Engl J Med. (2020) 383:2207–18. doi: 10.1056/NEJMoa2017699, PMID: 33264544

[168] Janjigian YY Kawazoe A Yañez P Li N Lonardi S Kolesnik O . The KEYNOTE-811 trial of dual PD-1 and HER2 blockade in HER2-positive gastric cancer. Nature. (2021) 600:727–30. doi: 10.1038/s41586-021-04161-3, PMID: 34912120 PMC8959470

[169] Kang YK Morita S Satoh T Ryu MH Chao Y Kato K . Exploration of predictors of benefit from nivolumab monotherapy for patients with pretreated advanced gastric and gastroesophageal junction cancer: *post hoc* sub analysis from the ATTRACTION-2 study. Gastric Cancer. (2022) 25:207–17. doi: 10.1007/s10120-021-01230-4, PMID: 34480657 PMC8732926

[170] Hartmann S Grandis JR . Treatment of head and neck cancer in the elderly. Expert Opin Pharmacother. (2016) 17:1903–21. doi: 10.1080/14656566.2016.1220540, PMID: 27643444

[171] Nederlof I Isaeva OI De Graaf M Gielen RCAM Bakker NAM Rolfes AL . Neoadjuvant nivolumab or nivolumab plus ipilimumab in early-stage triple-negative breast cancer: a phase 2 adaptive trial. Nat Med. (2024) 30:3223–35. doi: 10.1038/s41591-024-03249-3, PMID: 39284953 PMC11564107

[172] Denkert C Von Minckwitz G Darb-Esfahani S Lederer B Heppner BI Weber KE . Tumor-infiltrating lymphocytes and prognosis in different subtypes of breast cancer: a pooled analysis of 3771 patients treated with neoadjuvant therapy. Lancet Oncol. (2018) 19:40–50. doi: 10.1016/S1470-2045(17)30904-X, PMID: 29233559

[173] Zhu X Huang X Hu M Sun R Li J Wang H . A specific enterotype derived from gut microbiome of older individuals enables favorable responses to immune checkpoint blockade therapy. Cell Host Microbe. (2024) 32:489–505.e5. doi: 10.1016/j.chom.2024.03.002, PMID: 38513657

[174] Hansen SB Unal B Kuzu OF Saatcioglu F . Immunological facets of prostate cancer and the potential of immune checkpoint inhibition in disease management. Theranostics. (2024) 14:6913–34. doi: 10.7150/thno.100555, PMID: 39629128 PMC11610136

[175] Sato Y Silina K Van Den Broek M Hirahara K Yanagita M . The roles of tertiary lymphoid structures in chronic diseases. Nat Rev Nephrol. (2023) 19:525–37. doi: 10.1038/s41581-023-00706-z, PMID: 37046081 PMC10092939

[176] Li T Chen G Xiao Z Li B Zhong H Lin M . Surgical tumor-derived photothermal nanovaccine for personalized cancer therapy and prevention. Nano Lett. (2022) 22:3095–103. doi: 10.1021/acs.nanolett.2c00500, PMID: 35357839

[177] Hu Q Peng J Jiang L Li W Su Q Zhang J . Metformin as a senostatic drug enhances the anticancer efficacy of CDK4/6 inhibitor in head and neck squamous cell carcinoma. Cell Death Dis. (2020) 11:925. doi: 10.1038/s41419-020-03126-0, PMID: 33116117 PMC7595194

[178] Wang R Yu Z Sunchu B Shoaf J Dang I Zhao S . Rapamycin inhibits the secretory phenotype of senescent cells by a Nrf2-independent mechanism. Aging Cell. (2017) 16:564–74. doi: 10.1111/acel.12587, PMID: 28371119 PMC5418203

[179] Hurez V Dao V Liu A Pandeswara S Gelfond J Sun L . Chronic mTOR inhibition in mice with rapamycin alters T, B, myeloid, and innate lymphoid cells and gut flora and prolongs life of immune-deficient mice. Aging Cell. (2015) 14:945–56. doi: 10.1111/acel.12380, PMID: 26315673 PMC4693453

[180] Yan J Wang ZY Yang HZ Liu HZ Mi S Lv XX . Timing is critical for an effective anti-metastatic immunotherapy: The decisive role of IFNγ/STAT1-mediated activation of autophagy. PloS One. (2011) 6:e24705. doi: 10.1371/journal.pone.0024705, PMID: 21931823 PMC3172290

[181] Matacchione G Gurău F Silvestrini A Tiboni M Mancini L Valli D . Anti-SASP and anti-inflammatory activity of resveratrol, curcumin and β-caryophyllene association on human endothelial and monocytic cells. Biogerontology. (2021) 22:297–313. doi: 10.1007/s10522-021-09915-0, PMID: 33704623 PMC8084815

[182] Lim H Park H Kim HP . Effects of flavonoids on senescence-associated secretory phenotype formation from bleomycin-induced senescence in BJ fibroblasts. Biochem Pharmacol. (2015) 96:337–48. doi: 10.1016/j.bcp.2015.06.013, PMID: 26093063

[183] Alimbetov D Davis T Brook AJC Cox LS Faragher RGA Nurgozhin T . Suppression of the senescence-associated secretory phenotype (SASP) in human fibroblasts using small molecule inhibitors of p38 MAP kinase and MK2. Biogerontology. (2016) 17:305–15. doi: 10.1007/s10522-015-9610-z, PMID: 26400758 PMC4819486

[184] Liu X Liu X Mao W Guo Y Bai N Jin L . Tetrastigma polysaccharide reprogramming of tumor-associated macrophages via PPARγ signaling pathway to play antitumor activity in breast cancer. J Ethnopharmacol. (2023) 314:116645. doi: 10.1016/j.jep.2023.116645, PMID: 37196813

[185] Dorff TB Goldman B Pinski JK Mack PC Lara PN Van Veldhuizen PJ . Clinical and correlative results of SWOG S0354: A phase II trial of CNTO328 (siltuximab), a monoclonal antibody against interleukin-6, in chemotherapy-pretreated patients with castration-resistant prostate cancer. Clin Cancer Res. (2010) 16:3028–34. doi: 10.1158/1078-0432.CCR-09-3122, PMID: 20484019 PMC2898710

[186] San-Miguel J Bladé J Shpilberg O Grosicki S Maloisel F Min CK . Phase 2 randomized study of bortezomib-melphalan-prednisone with or without siltuximab (anti–IL-6) in multiple myeloma. Blood. (2014) 123:4136–42. doi: 10.1182/blood-2013-12-546374, PMID: 24833354 PMC4123433

[187] Angevin E Tabernero J Elez E Cohen SJ Bahleda R Van Laethem JL . A phase I/II, multiple-dose, dose-escalation study of siltuximab, an anti-interleukin-6 monoclonal antibody, in patients with advanced solid tumors. Clin Cancer Res. (2014) 20:2192–204. doi: 10.1158/1078-0432.CCR-13-2200, PMID: 24563479

[188] Ebersbach C Beier AMK Thomas C Erb HHH . Impact of STAT proteins in tumor progress and therapy resistance in advanced and metastasized prostate cancer. Cancers. (2021) 13:4854. doi: 10.3390/cancers13194854, PMID: 34638338 PMC8508518

[189] Wang Y Zhang Y . Prognostic role of interleukin-6 in renal cell carcinoma: A meta-analysis. Clin Transl Oncol. (2020) 22:835–43. doi: 10.1007/s12094-019-02192-x, PMID: 31410730

[190] Ando K Takahashi F Motojima S Nakashima K Kaneko N Hoshi K . Possible role for tocilizumab, an anti–interleukin-6 receptor antibody, in treating cancer cachexia. J Clin Oncol. (2013) 31:e69–72. doi: 10.1200/JCO.2012.44.2020, PMID: 23129740

[191] Hu Z Sui Q Jin X Shan G Huang Y Yi Y . IL6-STAT3-C/EBPβ-IL6 positive feedback loop in tumor-associated macrophages promotes the EMT and metastasis of lung adenocarcinoma. J Exp Clin Cancer Res. (2024) 43:63. doi: 10.1186/s13046-024-02989-x, PMID: 38424624 PMC10903044

[192] Dijkgraaf EM Santegoets SJAM Reyners AKL Goedemans R Wouters MCA Kenter GG . A phase I trial combining carboplatin/doxorubicin with tocilizumab, an anti-IL-6R monoclonal antibody, and interferon-α2b in patients with recurrent epithelial ovarian cancer. Ann Oncol. (2015) 26:2141–9. doi: 10.1093/annonc/mdv309, PMID: 26216383

[193] Haq ATA Yang PP Jin C Shih JH Chen LM Tseng HY . Immunotherapeutic IL-6R and targeting the MCT-1/IL-6/CXCL7/PD-L1 circuit prevent relapse and metastasis of triple-negative breast cancer. Theranostics. (2024) 14:2167–89. doi: 10.7150/thno.92922, PMID: 38505617 PMC10945351

[194] Bruchard M Mignot G Derangère V Chalmin F Chevriaux A Végran F . Chemotherapy-triggered cathepsin B release in myeloid-derived suppressor cells activates the Nlrp3 inflammasome and promotes tumor growth. Nat Med. (2013) 19:57–64. doi: 10.1038/nm.2999, PMID: 23202296

[195] Li W Xu J Zhao J Zhang R . Oxaliplatin and infliximab combination synergizes in inducing colon cancer regression. Med Sci Monit. (2017) 23:780–9. doi: 10.12659/MSM.901880, PMID: 28190020 PMC5319445

[196] Guo Y Xie F Liu X Ke S Chen J Zhao Y . Blockade of TNF-α/TNFR2 signaling suppresses colorectal cancer and enhances the efficacy of anti-PD1 immunotherapy by decreasing CCR8+T regulatory cells. J Mol Cell Biol. (2024) 16:mjad067. doi: 10.1093/jmcb/mjad067, PMID: 37935468 PMC11587560

[197] Yu H Lin L Zhang Z Zhang H Hu H . Targeting NF-κB pathway for the therapy of diseases: Mechanism and clinical study. Signal Transd Targ Ther. (2020) 5:209. doi: 10.1038/s41392-020-00312-6, PMID: 32958760 PMC7506548

[198] Pienta KJ Machiels JP Schrijvers D Alekseev B Shkolnik M Crabb SJ . Phase 2 study of carlumab (CNTO 888), a human monoclonal antibody against CC-chemokine ligand 2 (CCL2), in metastatic castration-resistant prostate cancer. Invest New Drugs. (2013) 31:760–8. doi: 10.1007/s10637-012-9869-8, PMID: 22907596

[199] Seya T Takeda Y Matsumoto M . A toll-like receptor 3 (TLR3) agonist ARNAX for therapeutic immunotherapy. Adv Drug Delivery Rev. (2019) 147:37–43. doi: 10.1016/j.addr.2019.07.008, PMID: 31302192

[200] Rodell CB Arlauckas SP Cuccarese MF Garris CS Li R Ahmed MS . TLR7/8-agonist-loaded nanoparticles promote the polarization of tumor-associated macrophages to enhance cancer immunotherapy. Nat BioMed Eng. (2018) 2:578–88. doi: 10.1038/s41551-018-0236-8, PMID: 31015631 PMC6192054

[201] Li W Lu L Lu J Wang X Yang C Jin J . cGAS-STING–mediated DNA sensing maintains CD8+ T cell stemness and promotes antitumor T cell therapy. Sci Transl Med. (2020) 12:eaay9013. doi: 10.1126/scitranslmed.aay9013, PMID: 32581136

[202] Li T Cheng H Yuan H Xu Q Shu C Zhang Y . Antitumor activity of cGAMP via stimulation of cGAS-cGAMP-STING-IRF3 mediated innate immune response. Sci Rep. (2016) 6:19049. doi: 10.1038/srep19049, PMID: 26754564 PMC4709567

[203] Liu Y Crowe WN Wang L Lu Y Petty WJ Habib AA . An inhalable nanoparticulate STING agonist synergizes with radiotherapy to confer long-term control of lung metastases. Nat Commun. (2019) 10:5108. doi: 10.1038/s41467-019-13094-5, PMID: 31704921 PMC6841721

[204] Xu N Palmer DC Robeson AC Shou P Bommiasamy H Laurie SJ . STING agonist promotes CAR T cell trafficking and persistence in breast cancer. J Exp Med. (2021) 218:e20200844. doi: 10.1084/jem.20200844, PMID: 33382402 PMC7780733

[205] Hu J Sánchez-Rivera FJ Wang Z Johnson GN Ho YJ Ganesh K . STING inhibits the reactivation of dormant metastasis in lung adenocarcinoma. Nature. (2023) 616:806–13. doi: 10.1038/s41586-023-05880-5, PMID: 36991128 PMC10569211

[206] Tezcan G Garanina EE Alsaadi M Gilazieva ZE Martinova EV Markelova MI . Therapeutic potential of pharmacological targeting NLRP3 inflammasome complex in cancer. Front Immunol. (2021) 11:607881. doi: 10.3389/fimmu.2020.607881, PMID: 33613529 PMC7887322

[207] Amo-Aparicio J Dominguez A Atif SM Dinarello A Azam T Alula KM . Pancreatic ductal adenocarcinoma cells regulate NLRP3 activation to generate a tolerogenic microenvironment. Cancer Res Commun. (2023) 3:1899–911. doi: 10.1158/2767-9764.CRC-23-0065, PMID: 37772994 PMC10510589

[208] Tengesdal IW Menon DR Osborne DG Neff CP Powers NE Gamboni F . Targeting tumor-derived NLRP3 reduces melanoma progression by limiting MDSCs expansion. Proc Natl Acad Sci. (2021) 118:e2000915118. doi: 10.1073/pnas.2000915118, PMID: 33649199 PMC7958415

[209] Theivanthiran B Evans KS DeVito NC Plebanek M Sturdivant M Wachsmuth LP . A tumor-intrinsic PD-L1/NLRP3 inflammasome signaling pathway drives resistance to anti–PD-1 immunotherapy. J Clin Invest. (2020) 130:2570–86. doi: 10.1172/JCI133055, PMID: 32017708 PMC7190922

[210] Bientinesi E Ristori S Lulli M Monti D . Quercetin induces senolysis of doxorubicin-induced senescent fibroblasts by reducing autophagy, preventing their pro-tumor effect on osteosarcoma cells. Mech Ageing Dev. (2024) 220:111957. doi: 10.1016/j.mad.2024.111957, PMID: 38909661

[211] Maggiorani D Le O Lisi V Landais S Moquin-Beaudry G Lavallée VP . Senescence drives immunotherapy resistance by inducing an immunosuppressive tumor microenvironment. Nat Commun. (2024) 15:2435. doi: 10.1038/s41467-024-46769-9, PMID: 38499573 PMC10948808

[212] Saleh T Carpenter VJ Tyutyunyk-Massey L Murray G Leverson JD Souers AJ . Clearance of therapy-induced senescent tumor cells by the senolytic ABT-263 via interference with BCL-XL–BAX interaction. Mol Oncol. (2020) 14:2504–19. doi: 10.1002/1878-0261.12761, PMID: 32652830 PMC7530780

[213] González-Gualda E Reinius MAV Macias D Morsli S Ge J Olan I . Treatment resistance to platinum-based chemotherapy in lung and ovarian cancer is driven by a targetable TGFβ Senescent secretome. Nat Aging. (2026) 6:368–392. Available online at: https://www.nature.com/articles/s43587-025-01054-2 (Accessed March 3, 2026). 10.1038/s43587-025-01054-2 PMC12920146 41634464

[214] Zhang J Zhang S Cheng C Zhu C Wang T Tang L . Targeting senescence with radioactive 223Ra/ba SAzymes enables senolytics-unlocked one-two punch strategy to boost anti-tumor immunotherapy. Biomaterials. (2025) 315:122915. doi: 10.1016/j.biomaterials.2024.122915, PMID: 39461062

[215] Noel M O’Reilly EM Wolpin BM Ryan DP Bullock AJ Britten CD . Phase 1b study of a small molecule antagonist of human chemokine (C-C motif) receptor 2 (PF-04136309) in combination with nab-paclitaxel/gemcitabine in first-line treatment of metastatic pancreatic ductal adenocarcinoma. Invest New Drugs. (2020) 38:800–11. doi: 10.1007/s10637-019-00830-3, PMID: 31297636 PMC7211198

[216] Nywening TM Wang-Gillam A Sanford DE Belt BA Panni RZ Cusworth BM . Targeting tumor-associated macrophages with CCR2 inhibition in combination with FOLFIRINOX in patients with borderline resectable and locally advanced pancreatic cancer: A single-center, open-label, dose-finding, non-randomized, phase 1b trial. Lancet Oncol. (2016) 17:651–62. doi: 10.1016/S1470-2045(16)00078-4, PMID: 27055731 PMC5407285

[217] Modak RV De Oliveira Rebola KG McClatchy J Mohammadhosseini M Damnernsawad A Kurtz SE . Targeting CCL2/CCR2 signaling overcomes MEK inhibitor resistance in acute myeloid leukemia. Clin Cancer Res. (2024) 30:2245–59. doi: 10.1158/1078-0432.CCR-23-2654, PMID: 38451486 PMC11094423

[218] Le DT Folprecht G Varghese AM Gutierrez M Noel M Trikalinos NA . Phase 1b/2 study of BMS-813160, a CCR2/5 dual antagonist, in combination with chemotherapy or nivolumab in patients with advanced pancreatic or colorectal cancer. J Immunother Cancer. (2026) 14:e011284. doi: 10.1136/jitc-2024-011284, PMID: 41571299 PMC12829384

[219] Shi C Bopp T Lo HW Tkaczuk K Lin J . Bazedoxifene as a potential cancer therapeutic agent targeting IL-6/GP130 signaling. Curr Oncol. (2024) 31:5737–51. doi: 10.3390/curroncol31100426, PMID: 39451730 PMC11505662

[220] Méndez−Clemente A Bravo−Cuellar A González−Ochoa S Santiago−Mercado M Palafox−Mariscal L Jave−Suárez L . Dual STAT−3 and IL−6R inhibition with stattic and tocilizumab decreases migration, invasion and proliferation of prostate cancer cells by targeting the IL−6/IL−6R/STAT−3 axis. Oncol Rep. (2022) 48:138. doi: 10.3892/or.2022.8349, PMID: 35703345 PMC9245073

[221] Dao KHT Gotlib J Deininger MMN Oh ST Cortes JE Collins RH . Efficacy of ruxolitinib in patients with chronic neutrophilic leukemia and atypical chronic myeloid leukemia. J Clin Oncol. (2020) 38:1006–18. doi: 10.1200/JCO.19.00895, PMID: 31880950 PMC7106977

[222] Febvre-James M Lecureur V Fardel O . Potent repression of C-reactive protein (CRP) expression by the JAK1/2 inhibitor ruxolitinib in inflammatory human hepatocytes. Inflammation Res. (2020) 69:51–62. doi: 10.1007/s00011-019-01293-1, PMID: 31654094

[223] Griso AB Acero-Riaguas L Castelo B Cebrián-Carretero JL Sastre-Perona A . Mechanisms of cisplatin resistance in HPV negative head and neck squamous cell carcinomas. Cells. (2022) 11:561. doi: 10.3390/cells11030561, PMID: 35159370 PMC8834318

[224] Padda SK Reckamp KL Koczywas M Neal JW Kawashima J Kong S . A phase 1b study of erlotinib and momelotinib for the treatment of EGFR-mutated, tyrosine kinase inhibitor-naive metastatic non-small cell lung cancer. Cancer Chemother Pharmacol. (2022) 89:105–15. doi: 10.1007/s00280-021-04369-0, PMID: 34773474 PMC8739290

[225] Bezzio C Vernero M Ribaldone DG Alimenti E Manes G Saibeni S . Cancer risk in patients treated with the JAK inhibitor tofacitinib: Systematic review and meta-analysis. Cancers. (2023) 15:2197. doi: 10.3390/cancers15082197, PMID: 37190126 PMC10136459

[226] Zhang Y Zhang Q Liu Q Dang H Gao S Wang W . Safety and efficacy of Jaktinib (a novel JAK inhibitor) in patients with myelofibrosis who are relapsed or refractory to ruxolitinib: A single-arm, open-label, phase 2, multicenter study. Am J Hematol. (2023) 98:1579–87. doi: 10.1002/ajh.27031, PMID: 37466271

[227] Novotny-Diermayr V Hart S Goh KC Cheong A Ong LC Hentze H . The oral HDAC inhibitor pracinostat (SB939) is efficacious and synergistic with the JAK2 inhibitor pacritinib (SB1518) in preclinical models of AML. Blood Cancer J. (2012) 2:e69–9. doi: 10.1038/bcj.2012.14, PMID: 22829971 PMC3366067

[228] Regenbogen T Chen L Trinkaus K Wang-Gillam A Tan BR Amin M . Pacritinib to inhibit JAK/STAT signaling in refractory metastatic colon and rectal cancer. J Gastrointest Oncol. (2017) 8:985–9. doi: 10.21037/jgo.2017.08.16, PMID: 29299358 PMC5750170

[229] Liao X Lochhead P Nishihara R Morikawa T Kuchiba A Yamauchi M . Aspirin use, tumor PIK3CA mutation, and colorectal-cancer survival. N Engl J Med. (2012) 367:1596–606. doi: 10.1056/NEJMoa1207756, PMID: 23094721 PMC3532946

[230] De Matteis R Flak MB Gonzalez-Nunez M Austin-Williams S Palmas F Colas RA . Aspirin activates resolution pathways to reprogram T cell and macrophage responses in colitis-associated colorectal cancer. Sci Adv. (2022) 8:eabl5420. doi: 10.1126/sciadv.abl5420, PMID: 35108049 PMC8809687

[231] Lin S Pan Y Xu C . Effects of aspirin on pancreatic cancer cells PANC-1 and its potential molecular mechanism. J BUON Off J Balk Union Oncol. (2020) 25:2449–55. 33277869

[232] Drew DA Cao Y Chan AT . Aspirin and colorectal cancer: The promise of precision chemoprevention. Nat Rev Cancer. (2016) 16:173–86. doi: 10.1038/nrc.2016.4, PMID: 26868177 PMC6741347

[233] Yiannakopoulou EC . Aspirin and NSAIDs for breast cancer chemoprevention. Eur J Cancer Prev. (2015) 24:416–21. doi: 10.1097/CEJ.0000000000000098, PMID: 25380191

[234] Pan P Huang YW Oshima K Yearsley M Zhang J Yu J . Could aspirin and diets high in fiber act synergistically to reduce the risk of colon cancer in humans? Int J Mol Sci. (2018) 19:166. doi: 10.3390/ijms19010166, PMID: 29316620 PMC5796115

[235] Alfonso L Ai G Spitale RC Bhat GJ . Molecular targets of aspirin and cancer prevention. Br J Cancer. (2014) 111:61–7. doi: 10.1038/bjc.2014.271, PMID: 24874482 PMC4090734

[236] Bibbins-Domingo K on behalf of the U.S . Preventive Services Task Force*. Aspirin use for the primary prevention of cardiovascular disease and colorectal cancer: U.S. preventive services task force recommendation statement. Ann Intern Med. (2016) 164:836–45. doi: 10.7326/M16-0577, PMID: 27064677

[237] Hsieh CC Chiu HH Wang CH Kuo CH . Aspirin modifies inflammatory mediators and metabolomic profiles and contributes to the suppression of obesity-associated breast cancer cell growth. Int J Mol Sci. (2020) 21:4652. doi: 10.3390/ijms21134652, PMID: 32629916 PMC7369784

[238] Liu Y Fang S Li X Feng J Du J Guo L . Aspirin inhibits LPS-induced macrophage activation via the NF-κB pathway. Sci Rep. (2017) 7:11549. doi: 10.1038/s41598-017-10720-4, PMID: 28912509 PMC5599518

[239] Qiu W Wang X Leibowitz B Liu H Barker N Okada H . Chemoprevention by nonsteroidal anti-inflammatory drugs eliminates oncogenic intestinal stem cells via SMAC-dependent apoptosis. Proc Natl Acad Sci. (2010) 107:20027–32. doi: 10.1073/pnas.1010430107, PMID: 21041628 PMC2993406

[240] Tinsley HN Gary BD Keeton AB Lu W Li Y Piazza GA . Inhibition of PDE5 by sulindac sulfide selectively induces apoptosis and attenuates oncogenic wnt/β-catenin–mediated transcription in human breast tumor cells. Cancer Prev Res (Phila Pa). (2011) 4:1275–84. doi: 10.1158/1940-6207.CAPR-11-0095, PMID: 21505183 PMC3151326

[241] Li N Xi Y Tinsley HN Gurpinar E Gary BD Zhu B . Sulindac selectively inhibits colon tumor cell growth by activating the cGMP/PKG pathway to suppress wnt/β-catenin signaling. Mol Cancer Ther. (2013) 12:1848–59. doi: 10.1158/1535-7163.MCT-13-0048, PMID: 23804703 PMC3800150

[242] Ciolino HP Bass SE MacDonald CJ Cheng RYS Yeh GC . Sulindac and its metabolites induce carcinogen metabolizing enzymes in human colon cancer cells. Int J Cancer. (2008) 122:990–8. doi: 10.1002/ijc.23218, PMID: 17985343

[243] Bian J Zhou Y Zhu W Ma K Chen J Huang S . Sulindac inhibits the proliferation and promotes apoptosis of laryngeal cancer cells by down-regulating the AKT/β-catenin pathway. In: Review. Research Square (2023). Available online at: https://www.researchsquare.com/article/rs-2816978/v1 (Accessed March 3, 2026).

[244] Li X Pathi SS Safe S . Sulindac sulfide inhibits colon cancer cell growth and downregulates specificity protein transcription factors. BMC Cancer. (2015) 15:974. doi: 10.1186/s12885-015-1956-8, PMID: 26673922 PMC4682223

[245] Bayraktar S Baghaki S Wu J Liu DD Gutierrez-Barrera AM Bevers TB . Biomarker modulation study of celecoxib for chemoprevention in women at increased risk for breast cancer: A phase II pilot study. Cancer Prev Res (Phila Pa). (2020) 13:795–802. doi: 10.1158/1940-6207.CAPR-20-0095, PMID: 32513785

[246] Qian X Yang H Ye Z Gao B Qian Z Ding Y . Celecoxib augments paclitaxel-induced immunogenic cell death in triple-negative breast cancer. ACS Nano. (2024) 18:15864–77. doi: 10.1021/acsnano.4c02947, PMID: 38829727

[247] Egashira I Takahashi-Yanaga F Nishida R Arioka M Igawa K Tomooka K . Celecoxib and 2,5-dimethylcelecoxib inhibit intestinal cancer growth by suppressing the wnt/β-catenin signaling pathway. Cancer Sci. (2017) 108:108–15. doi: 10.1111/cas.13106, PMID: 27761963 PMC5276826

[248] Samoudi A Abolhasani-Zadeh F Afgar A Jalilian E Zeinalynezhad H Langroudi L . Treatment of cancer-associated fibroblast-like cells with celecoxib enhances the anti-cancer T helper 1/treg responses in breast cancer. Naunyn Schmiedebergs Arch Pharmacol. (2025) 398:6099–112. doi: 10.1007/s00210-024-03641-3, PMID: 39652176

[249] Tołoczko-Iwaniuk N Dziemiańczyk-Pakieła D Nowaszewska BK Celińska-Janowicz K Miltyk W . Celecoxib in cancer therapy and prevention – review. Curr Drug Targ. (2019) 20:302–15. doi: 10.2174/1389450119666180803121737, PMID: 30073924

[250] Zuo C Hong Y Qiu X Yang D Liu N Sheng X . Celecoxib suppresses proliferation and metastasis of pancreatic cancer cells by down-regulating STAT3/NF-kB and L1CAM activities. Pancreatology. (2018) 18:328–33. doi: 10.1016/j.pan.2018.02.006, PMID: 29525378

[251] Jimeno A Amador ML Kulesza P Wang X Rubio-Viqueira B Zhang X . Assessment of celecoxib pharmacodynamics in pancreatic cancer. Mol Cancer Ther. (2006) 5:3240–7. doi: 10.1158/1535-7163.MCT-06-0565, PMID: 17172427

[252] Shen W Zhang X Du R Gao W Wang J Bao Y . Ibuprofen mediates histone modification to diminish cancer cell stemness properties via a COX2-dependent manner. Br J Cancer. (2020) 123:730–41. doi: 10.1038/s41416-020-0906-7, PMID: 32528119 PMC7463005

[253] Akrami H Moradi B Borzabadi Farahani D Mehdizadeh K . Ibuprofen reduces cell proliferation through inhibiting wnt/β catenin signaling pathway in gastric cancer stem cells. Cell Biol Int. (2018) 42:949–58. doi: 10.1002/cbin.10959, PMID: 29512256

[254] Greenspan EJ Madigan JP Boardman LA Rosenberg DW . Ibuprofen inhibits activation of nuclear β-catenin in human colon adenomas and induces the phosphorylation of GSK-3β. Cancer Prev Res (Phila Pa). (2011) 4:161–71. doi: 10.1158/1940-6207.CAPR-10-0021, PMID: 21205744 PMC3078769

[255] Todo M Horinaka M Tomosugi M Tanaka R Ikawa H Sowa Y . Ibuprofen enhances TRAIL-induced apoptosis through DR5 upregulation. Oncol Rep. (2013) 30:2379–84. doi: 10.3892/or.2013.2713, PMID: 24002210

[256] Andrews J Djakiew D Krygier S Andrews P . Superior effectiveness of ibuprofen compared with other NSAIDs for reducing the survival of human prostate cancer cells. Cancer Chemother Pharmacol. (2002) 50:277–84. doi: 10.1007/s00280-002-0485-8, PMID: 12357301

[257] Akrami H Aminzadeh S Fallahi H . Inhibitory effect of ibuprofen on tumor survival and angiogenesis in gastric cancer cell. Tumor Biol. (2015) 36:3237–43. doi: 10.1007/s13277-014-2952-3, PMID: 25542229

[258] Woo J Shin S Cho E Ryu D Garandeau D Chajra H . Senotherapeutic-like effect of silybum marianum flower extract revealed on human skin cells. PloS One. (2021) 16:e0260545. doi: 10.1371/journal.pone.0260545, PMID: 34914725 PMC8675675

[259] Chen L Diao L Yang Y Yi X Rodriguez BL Li Y . CD38-mediated immunosuppression as a mechanism of tumor cell escape from PD-1/PD-L1 blockade. Cancer Discov. (2018) 8:1156–75. doi: 10.1158/2159-8290.CD-17-1033, PMID: 30012853 PMC6205194

[260] Beavis PA Milenkovski N Henderson MA John LB Allard B Loi S . Adenosine receptor 2A blockade increases the efficacy of anti–PD-1 through enhanced antitumor T-cell responses. Cancer Immunol Res. (2015) 3:506–17. doi: 10.1158/2326-6066.CIR-14-0211, PMID: 25672397

[261] Han H Jain AD Truica MI Izquierdo-Ferrer J Anker JF Lysy B . Small-molecule MYC inhibitors suppress tumor growth and enhance immunotherapy. Cancer Cell. (2019) 36:483–497.e15. doi: 10.1016/j.ccell.2019.10.001, PMID: 31679823 PMC6939458

[262] Duffy MJ O’Grady S Tang M Crown J . MYC as a target for cancer treatment. Cancer Treat Rev. (2021) 94:102154. doi: 10.1016/j.ctrv.2021.102154, PMID: 33524794

[263] Gaballah AI Elsherbiny AA Sharaky M Hamed NO Raslan NA Almilaibary A . Dexamethasone-tamoxifen combination exerts synergistic therapeutic effects in tamoxifen-resistance breast cancer cells. Biosci Rep. (2024) 44:BSR20240367. doi: 10.1042/BSR20240367, PMID: 38864530 PMC11230869

[264] Clarisse D Offner F De Bosscher K . Latest perspectives on glucocorticoid-induced apoptosis and resistance in lymphoid Malignancies. Biochim Biophys Acta BBA - Rev Cancer. (2020) 1874:188430. doi: 10.1016/j.bbcan.2020.188430, PMID: 32950642

[265] Kim KN LaRiviere M Macduffie E White CA Jordan-Luft MM Anderson E . Use of glucocorticoids in patients with cancer: Potential benefits, harms, and practical considerations for clinical practice. Pract Radiat Oncol. (2023) 13:28–40. doi: 10.1016/j.prro.2022.07.003, PMID: 35917896

[266] Liu L Aleksandrowicz E Schönsiegel F Gröner D Bauer N Nwaeburu CC . Dexamethasone mediates pancreatic cancer progression by glucocorticoid receptor, TGFβ and JNK/AP-1. Cell Death Dis. (2017) 8:e3064–4. doi: 10.1038/cddis.2017.455, PMID: 28981109 PMC5680577

[267] Obradović MMS Hamelin B Manevski N Couto JP Sethi A Coissieux MM . Glucocorticoids promote breast cancer metastasis. Nature. (2019) 567:540–4. doi: 10.1038/s41586-019-1019-4, PMID: 30867597

[268] Herr I Pfitzenmaier J . Glucocorticoid use in prostate cancer and other solid tumors: Implications for effectiveness of cytotoxic treatment and metastases. Lancet Oncol. (2006) 7:425–30. doi: 10.1016/S1470-2045(06)70694-5, PMID: 16648047

[269] Mariotta M Perewusnnyk G Koechli OR Little JB Von Knebel Doeberitz M Mirimanoff RO . Dexamethasone-induced enhancement of resistance to ionizing radiation and chemotherapeutic agents in human tumor cells. Strahlenther Onkol. (1999) 175:392–6. doi: 10.1007/s000660050027, PMID: 10481771

[270] Luput L Sesarman A Porfire A Achim M Muntean D Casian T . Liposomal simvastatin sensitizes C26 murine colon carcinoma to the antitumor effects of liposomal 5-fluorouracil *in vivo*. Cancer Sci. (2020) 111:1344–56. doi: 10.1111/cas.14312, PMID: 31960547 PMC7156830

[271] Gao L Li L Zhang D Qiu J Qian J Liu H . TAPI-1 exhibits anti-tumor efficacy in human esophageal squamous cell carcinoma cells via suppression of NF-κB signaling pathway. Dig Dis Sci. (2024) 69:81–94. doi: 10.1007/s10620-023-08181-z, PMID: 38007701 PMC10787672

[272] Ruscetti M Morris JP Mezzadra R Russell J Leibold J Romesser PB . Senescence-induced vascular remodeling creates therapeutic vulnerabilities in pancreas cancer. Cell. (2020) 181:424–441.e21. doi: 10.1016/j.cell.2020.03.008, PMID: 32234521 PMC7278897

[273] Malekpour K Hazrati A Soudi S Hashemi SM . Mechanisms behind therapeutic potentials of mesenchymal stem cell mitochondria transfer/delivery. J Controlled Rel. (2023) 354:755–69. doi: 10.1016/j.jconrel.2023.01.059, PMID: 36706838

[274] Bai XF Ma JC Zhang C Chen Z He J Cheng SX . Click chemistry-assisted rejuvenation of aging T cells sensitizes aged mice to tumor immunotherapy. J Am Chem Soc. (2025) 147:16694–704. doi: 10.1021/jacs.5c05312, PMID: 40310278

